# Growth arrested live-attenuated *Leishmania infantum KHARON1* null mutants display cytokinesis defect and protective immunity in mice

**DOI:** 10.1038/s41598-018-30076-7

**Published:** 2018-08-02

**Authors:** Ana Maria Murta Santi, Juliane Sousa Lanza, Luiza Guimarães Tunes, Jacqueline Araújo Fiuza, Gaétan Roy, Alessandra da Silva Orfanó, Andréa Teixeira de Carvalho, Frédéric Frézard, André Luís Branco de Barros, Silvane Maria Fonseca Murta, Rubens Lima do Monte-Neto

**Affiliations:** 1 Instituto René Rachou - Fiocruz Minas, 31390-009 Belo Horizonte, Brazil; 2 Departamento de Fisiologia e Biofísica, Instituto de Ciências Biológicas, Universidade Federal de Minas Gerais, 31270-901 Belo Horizonte, Brazil; 3 Centre de Recherche en Infectiologie du Centre de Recherche du CHU de Québec et Département de Microbiologie, Infectiologie et Immunologie, Faculté de Médecine, Université Laval, Québec, G1V 4G2 Canada; 4 Departamento de Análises Clínicas e Toxicológicas, Faculdade de Farmácia, Universidade Federal de Minas Gerais, 31270-901 Belo Horizonte, Brazil

## Abstract

There is no safe and efficacious vaccine against human leishmaniasis available and live attenuated vaccines have been used as a prophylactic alternative against the disease. In order to obtain an attenuated *Leishmania* parasite for vaccine purposes, we generated *L. infantum KHARON1* (*KH1*) null mutants (Δ*Likh1*). This gene was previously associated with growth defects in *L. mexicana*. Δ*Likh1* was obtained and confirmed by PCR, qPCR and *Southern blot*. We also generate a *KH1* complemented line with the introduction of episomal copies of *KH1*. Although Δ*Likh1* promastigote forms exhibited a growth pattern similar to the wild-type line, they differ in morphology without affecting parasite viability. *L. infantum* KH1-deficient amastigotes were unable to sustain experimental infection in macrophages, forming multinucleate cells which was confirmed by *in vivo* attenuation phenotype. The cell cycle analysis of Δ*Likh1* amastigotes showed arrested cells at G_2_/M phase. Δ*Likh1-*immunized mice presented reduced parasite burden upon challenging with virulent *L. infantum*, when compared to naïve mice. An effect associated with increased *Li* SLA-specific IgG serum levels and IL-17 production. Thus, Δ*Likh1* parasites present an infective-attenuated phenotype due to a cytokinesis defect, whereas it induces immunity against visceral leishmaniasis in mouse model, being a candidate for antileishmanial vaccine purposes.

## Introduction

Leishmaniasis is a group of diseases caused by protozoa belonging to *Leishmania* genus (Ross, 1903). These diseases affect mainly poor and marginalized populations and recently, 97 countries, distributed in Africa, Asia, Americas, Europe and Oceania, reported the endemic transmission of leishmaniasis^1^. Leishmaniasis is a parasitic disease with the second highest mortality rate – about 20,000 to 30,000 annual deaths for visceral leishmaniasis (VL) – only behind malaria^2^. More than 58,000 cases of VL and 220,000 cases of cutaneous leishmaniasis (CL) are reported annually. However, due to the high underreporting rates, it is estimated that 12 million people are infected and that 2 million new cases of leishmaniasis occur annually^1,2^.

Decreased leishmaniasis incidence is a complex task that requires prevention and control measures to be taken in an integrated manner. Since the treatment of patients has little impact on the epidemiology of New World leishmaniasis, health education, vector and reservoir control are critical to reducing leishmaniasis cases^3,4^. In this scenario, the development of an effective and accessible human vaccine is the best strategy for the control of this serious disease. Despite the great effort employed in recent years, there is still no vaccine available for human use^5^. The main issues to be solved are the high cost, the antigenic robustness of the parasites and the great complexity of the host’s responses^6^.

It is known that vaccines composed of attenuated viruses and bacteria are considered gold standard against intracellular pathogens^7^. Studies using attenuated *Leishmania* have shown that this is a good strategy for producing a long-lasting protective immune response^8–11^. By inducing a subclinical infection, attenuated *Leishmania* elicit the generation of memory cells^5,12–14^. The infection with live attenuated *Leishmania* is similar to an infection with pathogenic parasites but has the advantages of preventing overt disease while also allowing the host immune system to interact with a large gamma of leishmanial essential antigens in the development of a protective immunity^15^.

The immune response in mammalian hosts infected by *Leishmania* involves a complex biochemical network recruiting different cell types such as, chemo- and cytokines, and therefore a thorough understanding of vaccine-induced immunity will further reveal the important mediators of a subsequent protective response^5,11–14,16–20^. In *Leishmania* infection, the host immune system is sorely compromised, mainly affecting the ability of T cells to proliferate, leading to defects in cytotoxic functions and IL-12 release^21^. For example, after an infection in susceptible mouse strains, such as BALB/c and Swiss, an induction of a Th2-type immune response characterized by secretion of anti-inflammatory or regulatory cytokines, IL-10, IL4, IL- 5, IL-6, can inhibit the production of IL-12 cytokine – inductor of macrophage leishmanicidal action – and down-regulate the activity of the enzyme inducible nitric oxide synthase (iNOS) so, the nitric oxide (NO) and IFN-γ production are often impaired, allowing parasites to disseminate and persist in the host tissue causing severe damage^6,22^.

Normally, a vaccination scheme that elicits both CD4 and CD8 T cell immune responses in a Th1-polarized type, with consequent production of pro-inflammatory cytokines, mainly IL-12, IFN-γ and TNF-α, can protect from further infection. However, the simplistic paradigm Th1 *vs*. Th2 is not always applicable to all forms of leishmaniasis caused by different *Leishmania* strains^23^. Other cell types, for example Th17 cells and regulatory T cells (Treg), play crucial roles in disease progression or remission, depending on: (I) *Leishmania* spp.; (II) host genetic variation and (III) phenotypic background, excluding any immunological conditions.

Recent publications on live attenuated vaccines using centrin knocked out *Leishmania donovani* parasites (Δ*LdCen*) have shown induction of Th1 type immune response in mice, hamsters, and dogs with protection from subsequent challenge by virulent *Leishmania* infection^24^. Δ*LdCen* induces higher expression of Th17 differentiation cytokines – IL-1β, IL-6, and TGF-β – in splenic dendritic cells and upregulates IL-17 production by splenocytes and both CD4 and CD8 T cells, resulting in protection against wild-type *L. donovani* challenge^25^.

The protein KHARON1 (KH1) was recently described through the characterization of a glucose transporter in *L. mexicana* (LmxGT1). KH1 is located at the base of the flagellum and associated with the cytoskeleton and the flagellar axoneme, therefore being necessary for targeting LmxGT1 from the flagellar pocket to the flagellum^26^. Promastigote forms of *L. mexicana* null mutants (Δ*Lmxkh1*) did not present any change in fitness, but Δ*Lmxkh1* amastigotes have a failure in cytokinesis, generating non-viable multinucleated forms. As a consequence, Δ*Lmxkh1* mutants were unable to sustain infection in BALB/c mice^27^.

Thus, in the present study, we performed *Kh1* gene disruption in *L. infantum*, the etiological agent of visceral leishmaniasis, and evaluated the phenotype of these mutants, regarding their growth rates *in vitro*, infectivity *in vitro* and *in vivo*, intracellular proliferation, morphology and cell-cycle. Furthermore, we evaluated a vaccination scheme in BALB/c mice immunized with Δ*Likh1* and challenged by *L. infantum* wild-type. The immunogenicity was assessed by tracking seroconversion and cytokine production in vaccinated mice.

## Results

### Confirmation of *KH1* deletion

**Figure 1:**
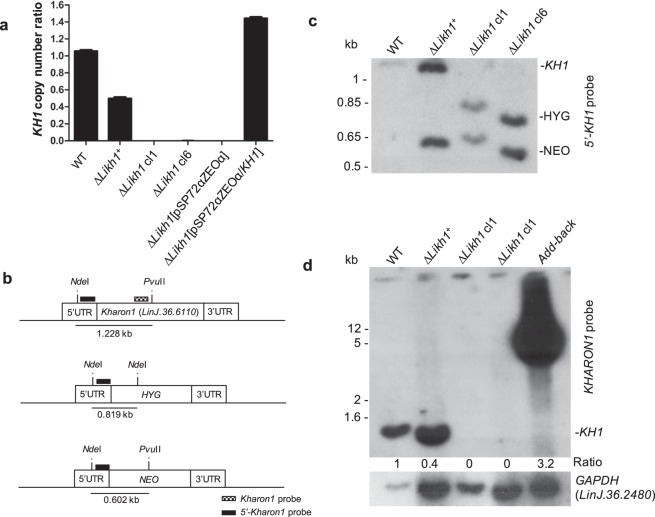
Confirmation of *KH1* deletion in *L. infantum*. (**a**) Comparison of the copy number of *KH1* gene in the genomic DNA of the mutant lines. The copy number was determined by quantitative real-time PCR relative to the housekeeping genes *GAPDH*, using the comparative C_T_ method (2^−ΔΔCT^ Method). (**b**) Schematic representation of *KH1* locus before and after the cassettes integration. The cleavage sites of *Nde*I and *Pvu*II restriction enzymes and the length of the generated fragments were represented. (**c**,**d**) *Southern blot* analysis performed with 10 µg of genomic DNA of *L. infantum* wild-type (WT), the mutants Δ*Likh1*^+^, Δ*Likh1* and Δ*Likh1*[pSP72αZEOα/*KH1*] were digested with *Nde*I and *Pvu*II restriction enzymes. The membranes were hybridized with *32P*-labelled probes, that reconize the *KH1* 5′UTR (**c**) or *Kh1* gene (**d**). The normalization was performed by calculating the *KH1/GAPDH* ratio. Similar results were obtained when normalizing with *DNA polymerase* as housekeeping gene. Exposures were cropped from original images available as supplementary material.

**Figure 2:**
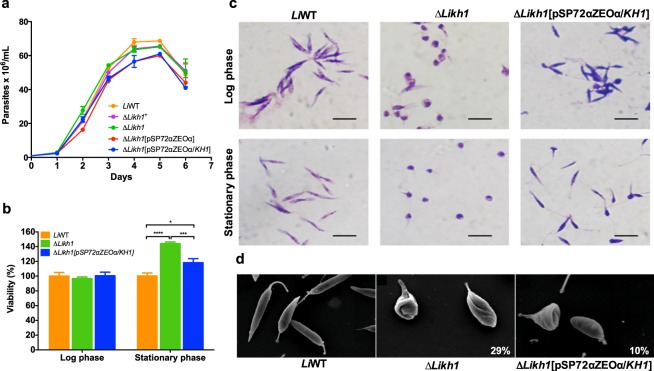
Growth profile, viability and morphology of axenically maintained promastigotes. (**a**) Growth curve of *L. infantum* promastigote lines of wild-type (WT) and mutants Δ*Likh1*^+^, Δ*Likh1*, Δ*Likh1*[pSP72αZEOα/*KH1*] and Δ*Likh1*[pSP72αZEOα]. The parasite number was automatically determined using the *Z1 Coulter® Particle Counter* (Beckman Coulter™). (**b**) Parasite viability at logarithmic (3rd day) and stationary (5th day) phase of growth as determined by MTT method. Columns represent the average values and the standard error of the mean from two independent experiments performed in quintuplicate analyzed by ANOVA followed by Tukey’s multicomparisons test. *p = 0.01; ***p = 0.001; ****p = 0.0001. (**c**) Optical microscopy of *L. infantum* WT, Δ*Likh1* and Δ*Likh1*[pSP72αZEOα/*KH1*] promastigotes stained with rapid panoptic at logarithmic and stationary phase of growth. Images were acquired using a *Nikon*^*®*^ *L810* camera coupled *Olympus BH2 Microscope System*. Bars: 10 μm. (**d**) Scanning electron microscopy-derived images of *L. infantum* WT, Δ*Likh1* and Δ*Likh1*[pSP72αZEOα/*KH1*] at log phase of growth. Electron micrographs were obtained using a scanning electron microscope JEOL JSM5600. Magnification: WT = 2500x; Δ*Likh1* and Δ*Likh1*[pSP72αZEOα/*KH1*] = 3000x. The right down percentages indicate the number of pear-like promastigotes (see supplementary Table S2).

In order to evaluate the correct integration of cassettes in mutant parasites, integration PCRs were performed (Supplementary Table S1). All clones presented fragments of expected size for the integration of both cassettes (Supplementary Fig. S1), showing that both cassettes are correctly integrated into the parasite’s genome. Furthermore, copy number of *KH1* gene was investigated by quantitative PCR in *L. infantum* Wild-Type (WT) and mutants. The pairs of primers *KH1*, *GAPDH* and *DNA polymerase* (Supplementary Table S1) amplified fragments of 105, 74 and 69 bp, respectively, and all of them had amplification efficiencies close to 100% (Supplementary Fig. S2). The normalization with both housekeeping genes (*GAPDH* and *DNA polymerase*) generated the same result. No amplification of *KH1* gene was observed in any of the Δ*Likh1* clones analyzed (Fig. 1a), while heterozygous knockout (Δ*Likh1::NEO/KH1*, here called Δ*Likh1*^+^) shows about half of the *KH1* copy number as compared to the WT line. The add-back line Δ*Likh1*[pSP72αZEOα/*KH1*] present a 1.5-fold increase on *KH1* copy number when compared to WT line (Fig. 1a). In addition, Southern blot was also performed in order to confirm *KH1* deletion (Fig. 1b,d). For both probes the expected band profile was obtained. The probe that recognizes the 5’UTR region (Fig. 2c) revealed a band of about 1.2 kb, corresponding to the *KH1* gene fragment only in the WT and Δ*Likh1*^+^ samples. Both Δ*Likh1* clones presented bands of expected size for the NEO and HYG fragments (819 and 602 bp respectively), and no bands corresponding to the *KH1* gene. Similarly, the probe that recognizes the *KH1* gene revealed a band of expected size in the WT and Δ*Likh1*^+^ samples, indicating the presence of the gene in these samples. No fragment was recognized in the DNA samples from Δ*Likh1* clones, confirming the success of *KH1* deletion. In the complemented (add-back) line, *KH1* probe revealed a very strong signal that is characteristic of extrachromosomal plasmid DNA migration in the gel, which corresponds to the construct pSP72αZEOα/*KH1* (Fig. 1d). Taken together, the results of conventional PCR, qPCR and Southern blot confirm the complete deletion of *KH1* in the mutants submitted to double replacements.

### Δ*Likh1* null mutants promastigote forms show no alteration in growth pattern

Null mutants (Δ*Likh1*) and the add-back line Δ*Likh1*[pSP72αZEOα/*KH1*] promastigote forms grew at similar rates compared to wild-type and the heterozygous knockouts (Δ*Likh1*^+^) parasites in axenic culture (Fig. 2a). Although no growth alteration was observed in terms of total parasite number, the morphology of Δ*Likh1* mutants differs from wild-type line. *L. infantum KHARON1* deficient mutants presented rounded parasites both at the log and stationary phase (Fig. 2c). Analysis by scanning electron microscopy, allowed us to observed that 30% of log-phase Δ*Likh1* promastigotes presented an alteration in cell shape at the posterior region, that we called pear-like form (Fig. 2d). Δ*Likh1*[pSP72αZEOα/*KH1*] parasites do not completely recover wild-type morphology (Fig. 2c). The characteristic pear-like promastigotes were still detected in 10% of cells during growth log-phase (Fig. 2d). Although we detected altered cellular shape, the mutant parasites remained viable. Indeed, Δ*Likh1* viability is increased in the stationary phase of axenic growth when compared with WT or Δ*Likh1*^+^ backgrounds (Fig. 2b). In addition, no difference was detected in glucose uptake by Δ*Likh1* null mutant parasites when compared to its wild-type counterpart (Supplementary Fig. S3). Furthermore, we evaluated the SbIII sensitivity in Δ*Likh1*, since glucose transport could be related to SbIII in *Leishmania*. However, no difference in sensitivity to SbIII was observed between Δ*Likh1* null mutant and wild-type lines, with SbIII IC_50_ at 184.9 µM and 179.8 µM respectively (Supplementary Fig. S4).

### Δ*Likh1* parasites are unable to sustain macrophage experimental infection

**Figure 3:**
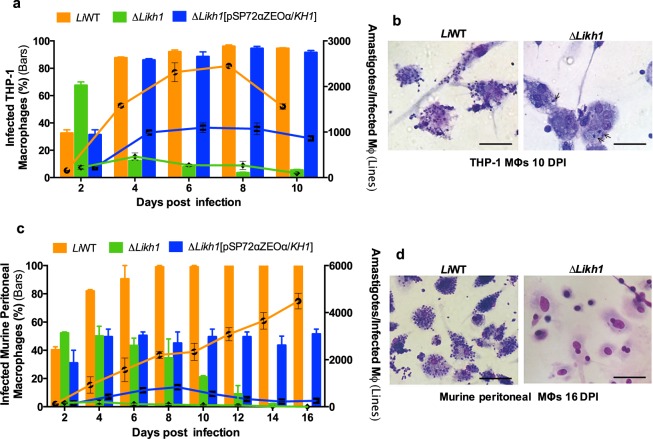
Experimental infection of THP-1 and murine peritoneal macrophages with Δ*Likh1*. THP-1 and murine peritoneal macrophages were infected with the following *L. infantum* lines: wild-type (WT), null mutant (Δ*Likh1*) and add-back (Δ*Likh1*[pSP72αZEOα/*KH1*]) and evaluated by 2 to 10 or 16 days post-infection (DPI). The columns represent the percentage of infected macrophages (MΦ) while the number of amastigote/infected macrophages are shown as overlapped lines in THP-1 (**a**) or murine-derived peritoneal macrophages (**c**). Optical photomicrographs were taken 10 and 16 DPI, respectively for THP-1 (**b**) and murine macrophages (**d**), infected with *L. infantum* WT and Δ*Likh1*. Arrows highlight intracellular amastigotes in Δ*Likh1-infected* THP-1 (**b**). Experimental infection was performed in macrophages adhered to 13 mm round sterile glass coverslips in 24-well cell culture plates (Sarstedt AG & Co., Nümbrecht, Germany), stained with panoptic rapid stained and mounted on microscope slides using Entellan® (Merk Millipore, Burlington, MA, USA). Images were acquired using a *Nikon*^*®*^ *L810* camera coupled with *Olympus BH2 Microscope System*.Bar: 50 µm.

**Figure 4:**
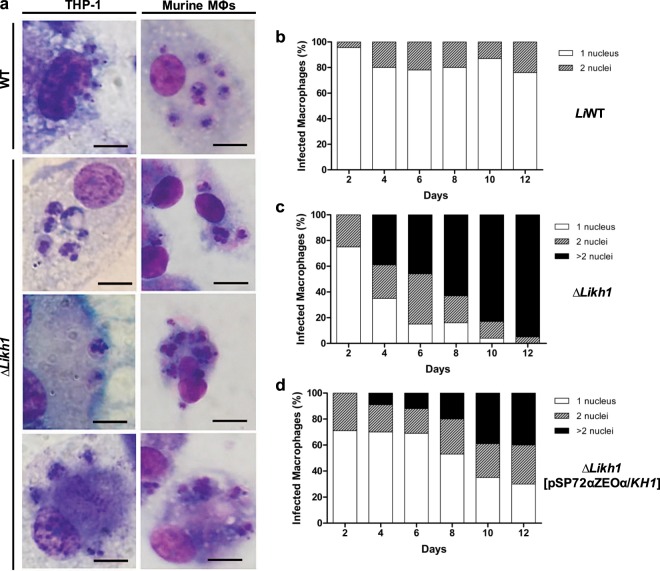
Qualitative and quantitative analysis of Δ*Likh1* multinucleated amastigotes. (**a**) Representative images of *Li*WT and Δ*Likh1* amastigotes infecting THP-1 or murine peritoneal macrophages, showing multinucleated Δ*Likh1* amastigotes. Bar represents 10 µm. Quantitative analysis of nuclei number in *Li*WT (**b**), Δ*Likh1* (**c**) and Δ*Likh1*[pSP72αZEOα/*KH1*] (**d**) intracellular amastigotes infecting murine peritoneal macrophages.

In order to evaluate infectivity and intracellular multiplication of Δ*Likh1* amastigotes, murine peritoneal macrophages or monocyte-derived human macrophages (THP-1 cell line) were infected with WT, Δ*Likh1* and Δ*Likh1*[pSP72αZEOα/*KH1*] parasites (Figs 3 and 4). *L. infantum* Δ*Likh1* amastigotes are able to infect macrophages and differentiate into amastigotes. However, these Δ*Likh1* mutants are unable to maintain infection in murine peritoneal and human-derived macrophages (Fig. 3). *KHARON1* null mutant parasites presented a low rate replication, not exceeding 300 parasites per 100 host cell for murine or human macrophages (Fig. 3a,c). Eight days post-infection, THP-1 macrophages presented 96% reduction in total number of Δ*Likh1*-infected cells and 94% reduction in amastigotes/100 macrophages ratio when compared to WT (Fig. 3a). Parasite clearance was observed after 16 days of infection in murine peritoneal macrophages infected with Δ*Likh1* mutants (Fig. 3c,d). The add-back cell line Δ*Likh1*[pSP72αZEOα/*KH1*] recovered the ability to replicate within murine or human macrophages, but infection rates were lower when compared with WT (Fig. 3a,c).

### Δ*LiKh1* possess multinucleated intracellular amastigotes

The quantitative evaluation of amastigote nuclei revealed the presence of multinucleated cells in Δ*Likh1* parasites. On the 12^th^-day post-infection, 95% of Δ*Likh1* parasites presented more than two nuclei (Fig. 4a,c). Add-back line also presented multinucleated cells, although in smaller proportions (Fig. 4d). At twelve days post infection, while 75% of the WT parasites had 1 nucleus (Fig. 4b), only 30% of *Likh1*[pSP72αZEOα/*KH1*] parasites had 1 nucleus and 40% had more than 2 nuclei (Fig. 4d).

### Δ*Likh1* amastigotes displayed a cytokinesis defect with cell-cycle arrest at G2/M

**Figure 5:**
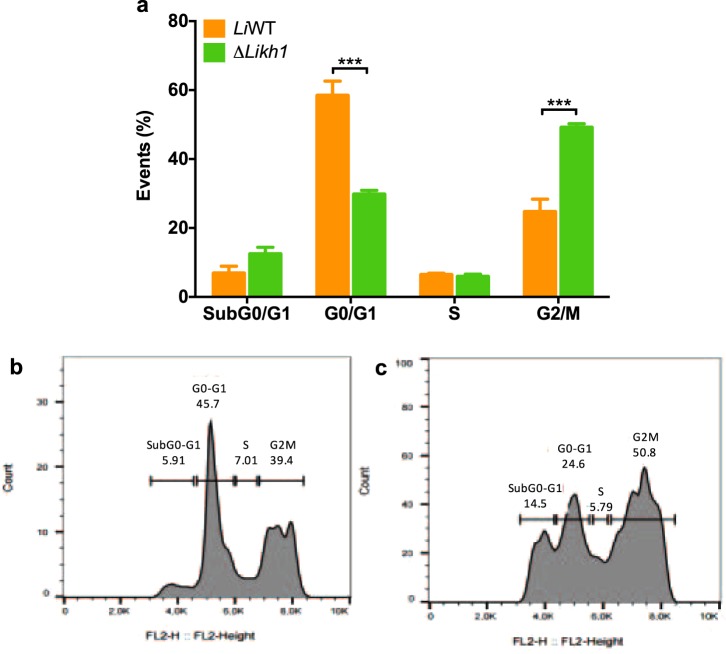
Cell cycle comparative analysis of *L. infantum* WT and *KH1* null mutants. *L. infantum* intracellular amastigotes were isolated from THP-1 infected macrophages and submitted to flow cytometry analysis after incubation with propidium iodide (PI). The percentage of cells in each phase is shown (**a**). The data represents the average of two independent experiments. Representative histograms show the DNA content of *L. infantum* WT (**b**), and *L. infantum* Δ*Likh1* amastigotes (**b**). Statistical analysis was performed based on ANOVA followed by Bonferroni’s multiple comparisons test (***p ≤ 0.001).

Cell cycle analysis using flow cytometry was performed in order to better understand the growth defects of Δ*Likh1* amastigotes compared to wild-type parasites. Δ*Likh1* parasites showed a reduced number of cells at G0/G1 phase which were arrested at G2/M phase when compared to the WT counterpart (Fig. 5). This phenotype is characteristic of a growth attenuated phenotype by decreasing their cellular cycling.

### Δ*Likh1* parasites show an attenuated virulence phenotype *in vivo*

**Figure 6:**
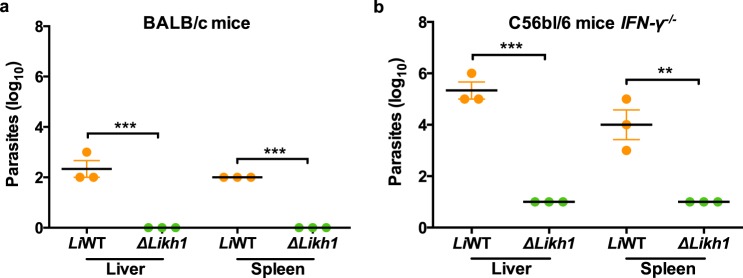
Parasite load in mice infected with *Li*WT and Δ*Likh1*. Parasite load was quantified by limiting dilution from samples isolated from liver and spleen of BALB/c (**a**) and *IFN-ɣ*^*−*/*−*^ C57BL/6 mice (**b**). BALB/c mice were infected with amastigotes (**a**) and C57BL/6 mice were infected with promastigotes forms (**b**). Samples were isolated on 15^th^-day post-infection. Statistical analysis was performed based on ANOVA followed by Bonferroni’s multiple comparisons test (**p ≤ 0.01; ***p ≤ 0.001).

To assess whether Δ*Likh1* parasites virulence was altered *in vivo*, different infection schemes were performed in BALB/c and immunocompromised C57BL/6 mice *IFN-γ*^*−*/*−*^. Amastigotes used in the *in vivo* infection assays were previously recovered from THP-1 macrophages. Infection of BALB/c mice with *L. infantum* WT amastigote forms were more successful when compared to infection using promastigote forms. *L. infantum* wild-type parasites were recovered from the spleen and liver, of all mice. However, the amastigote forms of Δ*Likh1* parasites were not able to infect BALB/c mice (Fig. 6a). In order to further investigate the infectivity of these parasites, promastigote forms of WT and Δ*Likh1* parasites were inoculated in *IFN-γ*^*−*/*−*^ C57BL/6 mice. Results of the limiting dilution assay (Fig. 6b) showed that Δ*Likh1* parasites were able to infect those mice, but lower Δ*Likh1* parasite number was recovered from spleen and liver of those mice when compared to its wild-type counterpart.

### Δ*Likh1* parasites induced seroconversion *in vivo* after priming by IV or SC routes

**Figure 7:**
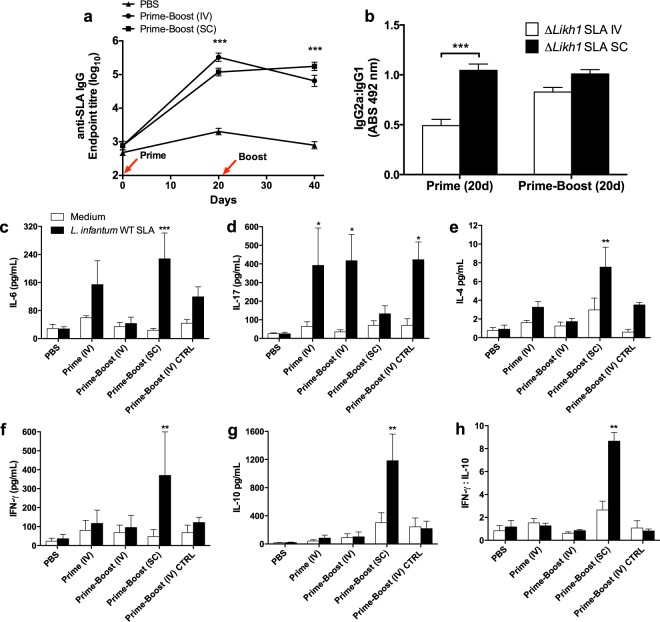
Humoral and cellular immune responses induced by different schemes of vaccination with Δ*Likh1*. Humoral immune response was evaluated by conventional ELISA measurement of serum levels of different isotypes of *Li*WT SLA-specific antibodies (IgG _total_, IgG_1_ and IgG_2a_). (**a**) Seroconversion of mice 20 days post-immunization with Δ*Likh1*. (**b**) IgG2a:IgG1 ratio between IV and SC prime-boosted groups. (**c**–**h**) Levels of cytokines in pg/mL in the supernatant of *Li*WT SLA-stimulated splenocytes. The concentration of *Li*WT SLA in the experiments was 25 μg/mL and splenocytes were stimulated for 72 h. CpG ODN at 5 μg/mL was used as a positive control of a Th1-response and α-MEM medium with 20% of FBS was used as negative control. Cytokines were measured by flow cytometry with the BD Cytometric Bead Array (CBA) Mouse Th1/Th2/Th17 Cytokine Kit (BD Biosciences, San Jose, CA, USA) following manufacturer instructions. The acquisition and data analyses were made in BD FACSVerse Instrument and FCAP Array Software v3. The detection limit of IL-2, IL-4, IL-6, IFN-γ, TNF, IL-17A and IL-10 cytokines were respectively 0.1; 0.03; 1.4; 0.5; 0.9; 0.8 and 16.8 pg/mL as well as cytokines secreted by *Li*WT SLA-stimulated splenocytes from immunized mice. Data are shown as means ± SE. Statistical analyses used One-way-ANOVA followed by Bonferroni’s multiple comparison test. ***p < 0,001. CpG ODN: CpG motifs oligodeoxynucleotides (Toll-like receptor 9 agonist); FBS: fetal bovine serum; IV: intravenous; SC: subcutaneous; SLA: soluble *Leishmania* antigens. Groups: 20 days after prime; 20 days after prime and boost; 20 days after challenging (vaccinated or not). PBS: *Li*WT- infected non-immunized mice; Prime-Boost (IV) CTRL: Δ*Likh1-*infected non-challenged (with *Li*WT) mice; Prime (IV): intravenously Δ*Likh1-*vaccinated mice once (prime) followed by *Li*WT challenge; Prime-Boost (IV): intravenously Δ*Likh1-*vaccinated mice twice (prime-boost) followed by *Li*WT challenge; Prime-Boost (SC): subcutaneously Δ*Likh1-*vaccinated mice twice (prime-boost) followed by *Li*WT challenge.

Twenty days after the last immunization of Balb/c mice with Δ*Likh1* parasites, by IV or SC route, the levels of Soluble *Leishmania* Antigens (SLA)-specific IgG’s subtypes in the serum were determined. Serum analysis showed significantly higher titers of SLA-specific IgG_Total_, IgG_1_ and IgG_2a_ in all immunized groups in comparison with the PBS group (*p* < 0,001) (see Supplementary Fig. S5), indicating a seroconversion at the first dose and a strong induction of *Leishmania*-specific antibody responses triggered by the vaccination schemes (Fig. 7a). The amount of SLA-specific IgG1 detected in the serum of mice immunized twice intravenously was greater than in those immunized subcutaneously (*p* < 0.001). The SC Prime-Boost vaccination scheme was able to induce significantly higher production of IgG2a than the IV scheme (*p* < 0.05). Although IgG2a:IgG1 ratio was higher for animals primed subcutaneously (*p* < 0.001) (Fig. 7b), after boosting this difference disappeared in comparison with IV immunized groups. Thus, even though SC immunization could polarize humoral response to the Th1 type, after a subsequent immunization SLA-specific IgG’s serum levels become similar in higher titers in comparison to IV Δ*Likh1-*immunized animals (Fig. 7b).

### Higher IL-17 secretion is associated with lower parasite burden in Δ*Likh1*-immunized BALB/c mice challenged by *Li*WT

**Figure 8:**
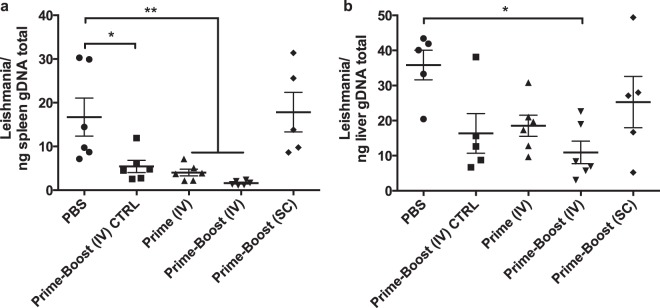
Parasite burden in liver and spleen of Δ*Likh1*-immunized mice followed by *Li*WT challenge. Δ*Likh1-*vaccinated animals (n = 6) were euthanized 20 days post-*L. infantum* challenge. Spleen (**a**) and liver (**b**) were removed, macerated and the DNA was extracted. The number of parasites in these organs was then determined by qPCR. Data are shown as median ± 95% CI. Before making statistical analysis, we performed a measurement of statistic dispersion by the IQR test (Interquartile range) in order to identify the outliers, but maintaining the minimum statistical accepted values which explains the variation in animal number per group.Analyses were done by Kruskall-Wallis, with Dunns posttest *p < 0.05; **p < 0.01. IV: intravenous; SC: subcutaneous; PBS: *Li*WT- infected non-immunized mice; Prime-Boost (IV) CTRL: Δ*Likh1-*infected non-challenged (with *Li*WT) mice; Prime (IV): intravenously Δ*Likh1-*vaccinated mice once (prime) followed by *Li*WT challenge; Prime-Boost (IV): intravenously Δ*Likh1-*vaccinated mice twice (prime-boost) followed by *Li*WT challenge; Prime-Boost (SC): subcutaneously Δ*Likh1-*vaccinated mice twice (prime-boost) followed by *Li*WT challenge.

The cytokines secreted by splenocytes of vaccinated mice were quantified 20 days after either last immunization or challenge with the virulent *Li*WT parasites. The amount of all cytokines but IL-17A in the culture supernatant after incubation for 72 h with SLA were significantly higher for the SC-vaccinated group when compared with unexposed splenocytes. This indicates that the scheme used induced a strong mixed Th1/Th2 immune response, in agreement with the humoral response (Fig. 7c–g). However, *Li*WT SLA-treated splenocytes presented higher IFN-γ:IL-10 ratio in SC-immunized mice (Fig. 7h), when compared with those maintained in free medium, indicating the polarization of the immune response to the Th1 type, which corroborates with the higher IgG2a:IgG1 ratio following prime in this group (Fig. 7b). Nevertheless, the induction of this differentiated immune response was not able to protect mice from a subsequent infection by *Li*WT, as demonstrated by liver and spleen parasite load, similar to the PBS-immunized and infected mice (Fig. 8a,b). In contrast, mice immunized IV either in prime or prime-boost scheme showed significantly lower parasite burden in liver and spleen (Fig. 8a,b), compared to PBS control group, but produced significantly higher levels of only IL-17A cytokine after SLA-stimulus (Fig. 7d). Considering a strong undifferentiated humoral response, this scenario highlights the importance of IL-17A production in protecting Δ*Likh1*-vaccinated mice against *Li*WT challenge It is worth mentioning that prime-boost (IV) Δ*Likh1*-immunized and non-challenged mice presented a significantly lower parasite load in comparison with the challenged ones (*p* < 0.001) (Fig. 8b). Curiously, it also produced higher levels of IL-17A cytokine. Although the Δ*Likh1* can colonize liver and spleen at a given level, the number of parasites in the organs is very low and in comparison with the *Li*WT parasites, the *KH1* knocked out parasites are much less infective *in vivo*, and this control of parasitism seems to be related to an effect of IL-17A cytokine and probably its promoting or differentiated cytokines, as well as the activation of Th17 responses.

## Discussion

In the present study we successfully disrupted *KHARON1* (*KH1*) gene in *L. infantum* based on gene replacement by conventional homologous recombination.

Promastigote forms of *L. infantum KH1* null mutants exhibited similar growth pattern to WT parasites, in accordance with previous studies of *KH1* gene deletion in *L. mexicana*^27^. However, morphology of Δ*Likh1* parasites was altered indicating a cytoskeleton impairment. Despite functional analysis, there is no known domain described for KH1 protein. However, sequence homology modeling revealed a C-terminal similarity with the villin headpiece domain (VHP – PF02209) that contains an F-actin binding site^28^. Villin (or gelsolin) is a calcium-regulated actin-binding protein that modulates cytoskeleton dynamics^29^. The similarity with villin VHP suggests that KH1 could also alter cytoskeleton organization in promastigotes of *L. infantum* and not only in amastigotes forms. This observation is also supported by the fact that KHARON in African Trypanosomes localizes at subpellicular microtubules and mitotic spindle, being associated with different components of the trypanosome cytoskeleton^30^.

As demonstrated in *L. mexicana*, KH1 is responsible for targeting the glucose transporter *Lmx*GT1 and was indirectly related to glucose transport in those parasites^26^. Here, we investigated the impact of the deletion of *KH1* on glucose uptake in *L. infantum* (Supplementary Fig. S3). It was found that the deletion of *KH1* in *L. infantum* did not interfere with glucose transport and this lack of phenotype is potentially a result of the presence of more than one *Leishmania* transporter capable of importing the hexoses glucose, mannose, fructose and galactose^31–34^. In *L. infantum* there is a locus encoding three glucose transporters isoforms were genes *LinJ.36.6540*, *LinJ.36.6550*, *LinJ.36.6560* code respectively for *Li*GT3, *Li*GT2 *and Li*GT1 (tritrypdb.org). Thus they are able to maintain normal glucose uptake in Δ*Likh1* parasites via bypass. Since energetic metabolism is also involved in the mechanism(s) of action of Sb-based drugs, such as beta-oxidation of fatty acids or glycolysis^35,36^, and because resistant-parasites present changes in fitness that result in lower glucose uptake and metabolism^37–39^, we evaluated the susceptibility of Δ*Likh1* to SbIII. We observed that Δ*Likh1* mutant exhibited sensitivity to trivalent antimony as the wild-type line (see Supplementary Fig. S4).

To evaluate the infectiveness of Δ*Likh1* amastigote forms, an *in vitro* experimental infection was performed in murine peritoneal and human-derived macrophages. Similar to the observed in *L. mexicana*^27^, *L. infantum KH1* deficient mutant were unable to maintain the infection within macrophages for more than 16 days *in vitro*. 10 to 12 days after infection, almost all parasites possess more than two nuclei, a phenotype that culminates with parasite’s death. We observed that Δ*Likh1* [pSP72αZEOα/*KH1*] add-back parasites do not completely recover the phenotype when compared to wild-type ones (Fig. 3), even though they present increased *KH1* copy number revealed by qPCR and Southern blot analyses (Fig. 1). When an episomal vector was used, it is possible that the daughter cells do not receive equal amounts of the vector, and the expression level of genes may vary from cell to cell, allowing the mosaicism observed^40^.

The presence of multinucleated forms suggests that although the parasites are able to perform karyokinesis, they are not able to finish cytokinesis. The phenotype presented by *L. infantum* Δ*kh1* parasites resembles those presented by *L*. *donovani* Centrin1 knockout (Δ*Ldcen*), in which there is no change in promastigote growth, and the amastigotes are multinucleated with cell-cycle arrest culminating with parasite death^41^. Like what was observed in the cell cycle of Δ*Ldcen* mutants^41^, *L. infantum* Δ*Likh1* also showed decreased cellular cycling, with retention at G2/M phase. This result corroborates the importance of KH1 protein for cell division in the amastigote forms of *L. infantum*.

Genetically modified live-attenuated *L. donovani* Δ*Ldcen* and Δ*Ldp27* has been described as vaccine candidates^10,42,43^, but also several targets have been implicated in loss of virulence upon disruption in different *Leishmania* species that often elicit protective immunity *in vivo* such as: silent information regulator (SIR2)^44^, A2^45^, arabino-1,4-lactone oxidase^9^ and biopterin transporter BT1^46,47^ in *L. donovani*; HSP70-II in *L. infantum*^48^; mitochondrial superoxide dismutase in *L. amazonensis*^49^; δ-amastins in *L. braziliensis*^50^ and cysteine protease B in *L. mexicana*^51^

To assess whether the attenuation phenotype of Δ*Likh1* mutants observed *in vitro* would also be observed *in vivo*, infections of BALB/c and *IFN-γ*^*−*/*−*^ C57BL/6 mice were performed. The fact that Δ*Likh1* parasites were not able to infect BALB/c mice and *IFN-γ*^*−*/*−*^ C57BL/6 mice infected with this strain had a much lower burden when compared to WT, shows that the knockout parasites are not effective in maintaining *in vivo* infection. Although some authors argue that parasites ability to generate a subclinical infection is critical for immunization, to result in lasting protection, such as in the case of leishmanization^15,52–56^, parasites may generate a protective immune response without, however, infecting the host, by the activation of dendritic cells and T lymphocytes IFN-ɣ production as demonstrated in immunizations with *L. tarentolae*^57^. The fact that Δ*Likh1* knockout did not infect mice efficiently, encourage the use of these parasites as a live attenuated vaccine as they can serve as a source of a broad range of immune potent antigens and induce a host protective immune response in animal models.

To confirm this hypothesis we performed immunization protocols in BALB/c mice to determine whether parasites were able to elicit humoral and cellular immune responses in the host, without causing disease, and determine if the administration route could affect the immunogenicity and the efficiency of protection from *Leishmania* infection. Both intravenous and subcutaneous priming of mice with the Δ*Likh1* parasites caused a significant increase in SLA-specific antibodies detected in mice serum, as a result of a strong humoral stimulation by either one or two-dose vaccination schemes. It is known that organisms exposed to whole or parts of parasites can develop a specific immunological memory against the harming agent that can be easily inferred by the presence of high titers of type G antibodies (IgG’s)^58^. The production of antigen-specific IgG_2a_ antibody subclass in mice may be related to the presence of the cytokine IFN-γ and to a Th1 response profile, generally more protective, whereas IgG_1_ is related to the presence of IL-4, associated with a Th2 type response and the increase in parasite load^59,60^. In our study, the increased production of IgG_2a_ subclass compared to IgG_1_ in the SC group in a first moment can be attributed to the administration route and was consistent with a Th1 response profile with the presence of large amount of IFN-γ. Conversely, not only did IgG production changed but also the Th2-type cytokines evaluated were upregulated in splenocytes after second dose and challenging. In this case, the mixed Th1/Th2 response may indicate a regulatory role rather than protective, as a mechanism to reverse the chronic inflammation generated. On the other hand, IV groups showed significantly higher amounts of SLA-specific IgG_1_, which could point to a stimulation of a more robust humoral response, correlated with a pro-inflammatory response noted only by the high production of IL-17A in detriment of all the other Th1 or Th2-type cytokines. Also, this immune response pattern was directly related to the decrease of parasite load in liver and spleen (in one or two Δ*Likh1* doses) of all IV-vaccinate mice. These findings strongly suggest that IL-17A and probably the Th17 cell activation plays a main role in mice protection. In accordance to our results, several studies using formulations with live attenuated vaccines or *Leishmania* isolated proteins alone or combined with immunoadjuvants, have demonstrated that the use of the isolated antigen led to the production of IgG_1_, but not IgG_2a_, whereas the use of an adjuvant – as TLR agonist like CpG’s ODNs – could drive the response to a more Th1 type, taken as required for protection against the infection^61^. Mice vaccination using recombinant polyprotein alone or combined with Th1-response immunostimulants results in a large production of both sub-classes of antigen-specific IgG_1_ and IgG_2a_ antibodies in a Th1/Th2 mixed response. Therefore, the literature indicates that the humoral immune response to vaccines is variable and does not show a simple relationship with the protection level, depending on the form the antigen is delivered, the associated adjuvant and the experimental model^57,62–64^. In our study we noticed that the administration route is also a critical factor to direct Th1 or Th2 polarized response in live attenuated vaccines^65^. The protective or susceptible immune mechanisms behind *Leishmania* infection are not clear and can vary according to host background and immunological state strain or parasite species, virulence, as well as the formulations, immunization scheme and administration route.

Here, the main mechanistic hint was the strong IL-17A cytokine elicitation triggered by vaccination with Δ*Likh1* parasites for host protection followed by virulent *Leishmania* infection. This cytokine and its lineage are largely described to play an important role in the clearance of pathogens as Gram-positive bacteria *Mycobacterium tuberculosis*, fungi, and parasites^66^ that require a massive inflammatory response which is not adequately dealt with Th1 or Th2 immunity leading to host protection. Furthermore, IL-17A was proven to be essential in host protection against *Trypanosoma cruzi*, during the acute phase of infection, being the hosts B cells the major sources of IL-17^61,67^. These studies highlight the importance of Th17 cells and IL-17A cytokine in controlling the development of intracellular pathogens. Thus, we can affirm that unique IV immunization with live-attenuated Δ*Likh1* parasites is able to seroconvert mice and induce a protective cell immune response with IL-17A release responsible for the protection of exposition and subsequent mice infection by virulent *L. infantum* opening new possibility for the development of a potent and effective vaccine against visceral leishmaniasis.

The results indicate that *KH1* deficient parasites obtained in this work are potential candidates for the development of an attenuated vaccine against leishmaniasis. Attenuated parasite-based vaccines are considered one of the most promising strategies for protection against Leishmaniasis, since these parasites simulate the natural course of *Leishmania* infection and allow the host immune system to contact a large antigenic repertoire^56,68^ activating antigen-presenting cells and generating memory cells^69^. These characteristics are crucial for the development of a lasting response^8,56^.

## Methods

### Parasites

Promastigote forms of *L. infantum* (MHOM/MA/67/ITMAP-263) were cultured in α-MEM medium (Gibco/Thermo Fisher Scientific, Waltham, MA, USA) supplemented with 10% fetal bovine serum, 5 μg/mL of hemin and 5 μM of biopterin, in pH 7 at 26 °C. Parasites were counted using the *Z1 Coulter® Particle Counter* (Beckman Coulter, Brea, CA, USA) and cultures were maintained by passing 1 × 10^6^ parasites to 5 mL of fresh medium twice a week. Growth curves were performed in 25 cm^2^ cell culture flasks by seeding 1 × 10^6^ parasite/mL and the number of parasites was determined daily – up to 6 days – by automatic counting using the Z1 Coulter Counter.

### Constructs for *KH1* knockout and complemented lines

The complete deletion of *KH1* (*LinJ.36.6110*) in *L. infantum* was performed by homologous recombination using linear cassettes that target the open reading frame (ORF) of *KH1* gene and contain the selection resistance markers neomycin phosphotransferase^70^ and hygromycin B phosphotransferase (HYG). The 5′UTR and 3′UTR regions flanking the open reading frame (ORF) of *LinJ.36.6110* were amplified by PCR from the *L. infantum* genomic DNA using specific primers (Supplementary Table S1). For amplification of the resistance markers, plasmids with the sequences of interest were used as templates. The constructs were obtained by fusion PCR approach and were cloned into the pGEM-T easy vector (Promega, Madison, WI, USA). In order to confirm the presence of expected insert, the plasmids were digested with restriction enzymes *Sal*I and *Xba*I, whose sites were pre-inserted into the primers for fusion PCR. The sequences were confirmed by Sanger sequencing. For plasmid complementation, *KH1* ORF was subcloned into vector pSP72αZEOα^71^ – harboring bleomycin-binding protein gene (ZEO), conferring resistance to zeocin – at *Sal*I and *Xba*I restriction sites. To confirm the presence of the correct insert, plasmids were digested with *Sal*I and *Xba*I.

### Transfections and parasite clone selection

Transfections were made by electroporation, performed using the Gene Pulser XCell^TM^ (BioRad, Hercules, CA, USA) by applying two sequential pulses of 1,500 V and 25 μF each as described previously^72,73^. The clonal selection was performed in semi-solid medium by addition of the antibiotics in the following concentrations: 600 µg/mL of Hygromicin (Invitrogen, Life Technologies), 80 µg/mL of Geneticin (G418) (Gibco/Thermo Fisher Scientific, Waltham, MA, USA) and 800 µg/mL Zeocin (Invitrogen/Thermo Fisher Scientific, Waltham, MA, USA). Mutant parasites were maintained in the presence of specific antibiotics for the resistance markers acquired at transfection. *KH1* gene deletion was confirmed by PCR, real-time qPCR and Southern blot. Conventional PCR was applied to verify the correct integration of *KH1* knockout cassette by using a reverse primer on HYG or NEO sequences and an external forward primer outside the cassette (Supplementary Fig. S1). Quantitative PCR was performed using genomic DNA to measure *KH1* gene copy number and Southern blot directly detected *KH1* by DNA probing hybridization.

### Quantitative real-time PCR

Genomic DNA was isolated from *L. infantum* WT, heterozygous knockout (Δ*Likh1*^+^), null mutant (Δ*Likh1*) and complemented add-back (Δ*Likh1* [pSP72αZEOα/*KH1*]) lines by phenol method according to the protocol previously described^74^. *KH1* gene was amplified from genomic DNA by real-time PCR in order to investigate *KH1* copy number. The housekeeping genes *GAPDH* (*LinJ.36.2480*) and DNA polymerase (*LinJ.16.1640*) were used as constitutive normalizer (Supplementary Table S1). Amplifications were performed using the Applied Biosystems 7500 thermocycler (Thermo Fisher Scientific, Waltham, MA, USA). PCR was carried out in a final volume of 20 µL of reaction mixture containing 10 pmol of forward and reverse primers, 1x SYBR GREEN reaction mix (Applied Biosystems/Thermo Fisher Scientific, Waltham, MA, USA) and 100 ng of template DNA. The PCR conditions were as follows: initial denaturation step at 95 °C for 10 min followed by 40 cycles of denaturation at 95 °C for 15 s and annealing/extension at 60 °C for 1 min. Fluorescence was measured at the end of each extension step. The copy number was determined using the comparative C_T_ method (2^−ΔΔCT^ Method).

### Southern blot

About 10 µg of genomic DNA from *L. infantum* WT, Δ*Likh1*^+^, Δ*Likh1* and Δ*Likh1*[pSP72αZEOα/*KH1*] were digested with restriction enzymes *Pvu*II and *Nde*I (Promega, Madison, WI, USA). The fragments were separated by electrophoresis on 1% agarose gel and transferred by capillarity onto nylon membranes (Hybond-N+, GE Healthcare Life Sciences, Malborough, MA, USA). The blots were hybridized with [α-^32^P]dCTP labeled *KH1* and 5′UTR *KH1* gene probes amplified with specific primers (Supplementary Table S1) according to standard protocols^70^. GAPDH probe was used as load control. Densitometric analyzes and semi-quantification were performed using *ImageJ* software (imagej.nih.gov/ij).

### Promastigote viability assay

*Leishmania* promastigote viability was assessed using the classical 3-(4,5-dimethylthiazol-2-yl)-2,5 diphenyltetrazolium bromide (MTT) method^75^. Briefly, promastigote forms at 3^rd^ and 5^th^ days of culture (log and stationary phase of growth, respectively) were washed in Hepes-NaCl solution (20 mM Hepes, 0.15 M NaCl, 10 mM glucose, pH 7.2), and cell suspension was seeded in 96-well flat bottom plates (5 × 10^6^ parasites/well in 0.2 mL final volume). Promastigote forms of *L. infantum* WT, Δ*Likh1*^+^, Δ*Likh1* and Δ*Likh1*[pSP72αZEOα/*KH1*] were exposed to 0.5 mg/mL of MTT and incubated 4 h at 37 °C in humid atmosphere containing 5% CO_2_. The plates were centrifuged, the supernatant was aspirated and added 0.1 mL of DMSO to dissolve formazan crystals. MTT formazan product was measured spectrophotometrically at 570 nm and cell viability calculated relative to WT growth.

### Ultrastructural evaluation

Scanning Electron Microscopy (SEM) was used to evaluate the morphological differences between *L. infantum* WT, Δ*Likh1*^+^, Δ*Likh1* and Δ*Likh1*[pSP72αZEOα/*KH1*]. The parasites were fixed using a solution of paraformaldehyde 4%, sodium cacodilate 0.1 M, glutaraldehyde 1% and calcium chloride 1 mM for 2 h at room temperature. The samples were then attached to a coverslip treated with Poly L-Lysine (SigmaAldrich, St. Louis, MI, USA) for 30 min in a wet chamber. After parasites adhesion onto coverslips, the samples were post-fixed in osmium tetroxide 1% for 2 h. Samples were dehydrated in a graded series of acetone, critical point dried with CO_2_ (Emitech K850), sputter coated with gold particles (Emitech K550), mounted on stubs and analyzed with Scanning Electron Microscopy Jeol - JSM5600.

### Δ*Likh1* cell cycle

To perform intracellular amastigote purification, infected THP-1 macrophages were collected on the 4^th^ day of infection using cell scrapper (Nunc™, Nalge Nunc International, Penfield, NY, USA). Macrophages were passed 30 times through a 27-gauge needle for macrophage disruption and amastigote release. Amastigotes were separated from the cell fragments by differential centrifugation: first centrifugation at 134 × *g* for 5 min was performed in order to remove macrophage fragments. The supernatant with amastigotes was collected and washed 4 times with Hepes-NaCl solution (20 mM Hepes, 0.15 M NaCl, 10 mM glucose, pH 7.2) and centrifuged at 1200 × *g* for 5 min. Amastigotes were counted and suspended in hypotonic fluorochromic solution (HFS) [0.1% sodium citrate, 0.1% triton × 100, 50 μg/mL propidium iodide (PI)]. Samples were incubated for 4 h at 4 °C, protected from light. The readings were performed on flow cytometer (FacsCalibur^®^, BD, Franklin Lakes, NJ, USA), acquiring 50,000 events/sample. Data analysis was performed using FlowJo^®^ software (FlowJo, LLC).

### Experimental infection

THP-1 human monocytic lineage were cultured in complete RPMI-1640 medium (supplemented 10% fetal bovine serum, 2 mM glutamine, 100 U/mL penicillin and 100 μg/mL streptomycin) and differentiated into macrophages by the adding 20 ng/mL phorbol myristate acetate (PMA) in culture. Cells (3 × 10^5^ macrophages/well) were seeded in 24 well cell culture plate containing 13 mm round coverslips and incubated for 72 h at 37 °C in a humid atmosphere containing 5% CO_2_. Similarly, the murine peritoneal macrophages elicited by thioglycollate were collected by washing the peritoneal cavity of BALB/c mice with RPMI-1640 medium and seeded as above. Macrophages (THP-1 and murine-derived ones) were incubated with parasites in the stationary phase (10 *Leishmania* per macrophage). After 3 h, uninternalized parasites were removed by successive washes. Parasite’s infectivity was monitored every 48 h. The coverslips were collected and stained by rapid panoptic method^76^. Briefly, the coverslips containing adhered infected macrophages were immersed in fixative solution (fast green in methanol) for 10 s, followed by the same procedure using stain solution 1 (eosin G in phosphate buffer – red colour) and stain solution 2 (thiazine dye in phosphate buffer – blue color). The coverslips were washed, dried and mounted in microscopy slides using Entellan® (Merk Millipore, Burlington, MA, USA). The infection was quantified by counting intracellular amastigotes using *ImageJ* software (imagej.nih.gov/ij).

### Mice infection and parasite recovery

To evaluate the infectivity of the knockout mutants, BALB/c mice and *IFN-γ*^*−*/*−*^ C57BL/6 mice were infected with WT and Δ*Likh1* parasites. BALB/c mice were infected with amastigotes while C57BL/6 mice were infected with promastigotes forms. Amastigotes were previously recovered from THP-1 macrophages, as described in previous section Δ*Likh1* cell cycle. In order to recover the parasites, spleens and livers of infected animals were collected 15 days after infection. Limiting dilution assays were performed as proposed by Titus *et al*.^77^. The organs of infected mice were processed using cell strainer (Corning, Corning, NY, USA) and the samples centrifuged at 134 × *g* for 10 min. The infected cells obtained from liver and spleen maceration were not lysed but suspended in α-MEM medium and added to the first wells of 96-well plates. From that initial concentration of cells, successive dilutions were made to the 12^th^ well, using 10x dilution factor. Plates were incubated for 15 days at 26 °C and then evaluated for parasite growth.

### Immunization and challenging assays

BALB/c female mice aged between 4–6 weeks old were divided into groups of at least 6 animals and immunized with 10^7^ Δ*Likh1* null mutant parasites, via tail vein (IV) in one or two dose schemes (Prime or Prime-Boost). A group receiving the same dose of parasites, but subcutaneously (SC) were also tested and the interval between the prime and boost were 20 days for all groups. Blood was collected at day 20 after priming and boosting, and serum was stored at −20 °C until analysis of IgG’s levels by ELISA. 20 days after the last immunization animals were infected with 10^7^ *Li*WT virulent parasites given intravenously. 20 days after challenging animals were euthanized and evaluated for immunogenicity and protective effects induced by the vaccination schemes. The groups were established as following: PBS: *Li*WT- infected non-immunized mice; Prime-Boost (IV) CTRL: Δ*Likh1-*infected non-challenged (with *Li*WT) mice; Prime (IV): intravenously Δ*Likh1-*vaccinated mice once (prime) followed by *Li*WT challenge; Prime-Boost (IV): intravenously Δ*Likh1-*vaccinated mice twice (prime-boost) followed by *Li*WT challenge; Prime-Boost (SC): subcutaneously Δ*Likh1-*vaccinated mice twice (prime-boost) followed by *Li*WT challenge.

### Soluble Antigen (SLA) preparation and antibody response

SLA was prepared as previously described^78^. Stationary-phase parasites were recovered, washed three times with PBS and suspended in lysis buffer containing 50 mM of Tris-HCl and 5 mM of EDTA at pH 7 (1 mL for every 10^9^ cells). Parasites were then frozen and thawed 10 times in liquid nitrogen and water bath at 40 °C followed by 3 cycles of pulsed sonication, 20 seconds each, at 40 W (Q500 Ultrasonicator, QSonica^®^, Newtown, CT, USA). The preparation was centrifuged at 5000 × *g* for 20 minutes, suspended in the same buffer and the total amount of protein was quantified by spectrophotometry at ABS of 200 and 260 nm (Synergy 2 reader, BioTek Instruments, Inc., Winooski, VT, USA) with the predefined application of protein measurements in the Take3 Section of Gen5^TM^ software v3.03. For IgG specific Ab responses, conventional ELISA method was used. Serum was analyzed for Th2-associated Ab, IgG1, and for Th1-associated Ab, IgG2a. Briefly, 96-wells plates were coated with 100 μL of a 25 μg/mL *Li*WT SLA solution and incubated at 4 °C overnight. After washes with PBS Tween 20 at 0.02% (PBST), serial dilutions of mice serums were added starting from 1:50. Peroxidase-conjugated rabbit anti-mouse IgG Total, IgG1 and IgG2 antibodies were added at a 1:5000 dilution for 1 h. The reaction was performed by addition of o-phenylenediamine and H_2_O_2_ (SigmaAldrich, St. Louis, MI, USA). After 15 min, the reaction was stopped by the addition of 100 μL of H_2_SO_4_ at 0.5 M. Absorbance at 492 nm. Cut-off values were calculated using the mean average OD of 60 negative samples plus 2 standard deviation and the endpoint IgG’s titers were calculated as previously described^79^.

### Cytokine production assessments

Twenty days after priming (for one-dose vaccinated mice), boosting (for two-doses vaccinated mice) and challenging, 6 mice per group were euthanized, spleens were harvested and splenocytes were isolated as previously described^80^. Singled-cells preparations were adjusted for 10^7^/mL and plated in 96-well flat-bottom tissue culture plate (Costar, Cambridge, MA, USA). Splenocytes were cultured at 37 °C, in 5%CO_2_ atmosphere in presence of 25 μg/mL SLAs, CpG-ODN (as a Th1-response inducer control) or supplemented medium (as a negative control). After 72 h incubation plates were centrifuged at 1000 × *g* for 10 min and supernatants were collected and analyzed for multiple cytokine production by flow cytometry with the BD Cytometric Bead Array (CBA) Mouse Th1/Th2/Th17 Cytokine Kit (BD Biosciences, San Jose, CA, USA) following manufacturer instructions. The acquisition and data analyses were made in BD FACSVerse Instrument and FCAP Array Software v3. The limit of detection of IL-2, IL-4, IL-6, IFN-γ, TNF, IL-17A and IL-10 cytokines were respectively 0,1; 0,03; 1,4; 0,5; 0,9; 0,8 and 16,8 pg/mL.

### Evaluation of parasite burden

Twenty days after challenging, 6 mice per group were euthanized and their liver and spleen were collected in separated tubes containing PBS. The organs were macerated and an aliquot was added in a microtube containing lysis buffer and Proteinase K, vortexed and incubated at 56 °C overnight. After incubation, DNA was extracted with Illustra tissue & cells genomic Prep Mini Spin Kit (GE, Healthcare Lifesciences, Malborough, MA, USA), according to the manufacturer’s instructions. The gDNA concentrations were measured by spectrophotometry (Abs at 280/260 nm) and adjusted to 10 ng/μL. 5 μL of each sample was used to a final volume of 20 μL per reaction that included ultrapure water, SYBR Green/ROX qPCR Master Mix (Thermo Fisher Scientific, Waltham, MA, USA), sense (forward, 5′-CCTATTTTACACCAACCCCCAGT-3′, and antisense primers (reverse, 5′-GGGTAGGGGCGTTC TGCGAAA-3′), constructed for amplification of the mini-circle region present in the kinetoplast DNA (kDNA) of approximately 120 bp. The standard curve was constructed with serial dilutions of the known concentrations of gDNA of *L. infantum*, extracted from promastigote culture. Ultrapure water was used as negative control. The amplification protocol included an annealing temperature and extension of 60 °C, with melting curve construction, on the ABI 7500 equipment (Applied Biosystems, Thermo Fisher Scientific, Waltham, MA, USA) and the analysis were made using the 7500 System Software. The standard curve correlated the known amount of *L. infantum* gDNA with the cycle threshold (Ct) given by the amplification of each point of the curve. A linear regression analysis was applied to the curve and the amount of parasite gDNA in mass of each sample was obtained by interpolation in standard curve and converted into number of parasites, considering that each genomic DNA of *L. infantum* has about 0.1 pg of mass^81^. Before making data variance analysis, we performed a measurement of statistic dispersion by the IQR test (Interquartile range) in order to identify the outliers, but maintaining the minimum statistical accepted values which explains the variation in animal number per group.

### Ethical statement

All procedures using animal models were previously approved by the institutional animal care committee: Ethics Committee for the Use of Animals – Oswaldo Cruz Foundation – CEUA/Fiocruz. All methods were performed in accordance with the guidelines and regulations from the National Council for the Control of Animal Experimentation – CONCEA and Canadian Council on Animal Care – CCAC, under license: CEUA #LW-28/14.

### Statistical analysis

Statistical analyzes were performed with Kruskal Wallis non-parametric test and Dunns posttest (or Mann Whitney test) for non-normal distribution data; and the One-way ANOVA followed by Bonferroni’s multiple comparisons test (or students’ t test) for normal distribution data.

## Electronic supplementary material

Supplementary Information
