# Supplementary material for: Growth arrested live-attenuated Leishmania infantum KHARON1 null mutants display cytokinesis defect and protective immunity in mice
Source: Sci Rep. 2018 Aug 2;8:11627. doi: 10.1038/s41598-018-30076-7 (PMC6072785; doi:10.1038/s41598-018-30076-7)

## **SUPPLEMENTARY INFORMATION**

**Growth arrested live-attenuated *Leishmania infantum KHARON1* null mutants display cytokinesis defect and protective immunity in mice**

Ana Maria Murta Santi^1^, Juliane Sousa Lanza^2^, Luiza Guimarães Tunes^1^, Jacqueline Araújo Fiuza^1^, Gaétan Roy^3^, Alessandra da Silva Orfanó^1^, Andréa Teixeira de Carvalho^1^, Frédéric Frézard^2^, André Luís Branco de Barros^4^, Silvane Maria Fonseca Murta^1^, Rubens Lima do Monte-Neto^1*^

^1^ Instituto René Rachou – FIOCRUZ MINAS, Belo Horizonte, MG, Brasil

^2^ Departamento de Fisiologia e Biofísica, Instituto de Ciências Biológicas, Universidade Federal de Minas Gerais, Belo Horizonte, MG, Brasil

^3^ Centre de Recherche en Infectiologie du Centre de Recherche du CHU de Québec et Département de Microbiologie, Infectiologie et Immunologie, Faculté de Médecine, Université Laval, Québec, QC, Canada

^4^ Departamento de Análises Clínicas e Toxicológicas, Faculdade de Farmácia, Universidade Federal de Minas Gerais, Belo Horizonte, MG, Brasil

*Corresponding author: Rubens L. Monte-Neto, e-mail: rubens.neto@minas.fiocruz.br

| TEST | GENE | NAME | | SEQUENCE (5’⭢3’) | Tm (°C) | FRAGMENT SIZE (bp) |
| --- | --- | --- | --- | --- | --- | --- |
| Fusion PCR | 5'UTR of *KH1* | 1 | XbaI.5'-*KH1*_Fw | TCTAGAGTATCGTCCGAGTTCCCTTTC | 58,8 |  |
|  |  | 2 | degHYG_5'-*KH1*_Rev | GTGAGTTCAGGCTTTTTCATCGTGGAAGTGCTCGAGACGG | 72 | 554 |
|  |  | 3 | degNEO_5'-*KH1*_Rev | AATCCATCTTGTTCAATCATCGTGGAAGTGCTCGAGACGG | 72 |  |
|  | 3'UTR of *KH1* | 4 | 3'*KH1*_Fw | GAAGCAGCACCACCCCTGGG | 63,6 | 528 |
|  |  | 5 | Sal.3'-*KH1*_Rev | GTCGACCTGTTTGCTGCGCTGTTTAT | 61,7 |  |
|  | HYG | 6 | HYG_Fw | ATGAAAAAGCCTGAACTCACCGCG | 60,2 | 1.025 |
|  |  | 7 | deg3'*KH1*_HYG_Rev | CCCAGGGGTGGTGCTGCTTCTCATCGATGATGGGGATCT | 71,9 |  |
|  | NEO | 8 | NEO_Fw | ATGATTGAACAAGATGGATTGCACGC | 58,4 | 794 |
|  |  | 9 | deg3'*KH1*_NEO_Rev | CCCAGGGGTGGTGCTGCTTCTCAGAAGAACTCGTCAAGAA | 69,2 |  |
| Integration PCR | 5'UTR::NEO and 5'UTR:HYG | 10 | 5'out*KH1*_Fw | TTTGGTGCTGGTGAGTGTAG | 55,7 | 1.600 and  1.476 |
|  |  | 11 | NEO_mid_Rev | GCCAACGCTATGTCCTGATA | 57,2 |  |
|  |  | 12 | HYG_mid_Rev | CTGACGGTGTCGTCCATAAC | 57,4 |  |
| qPCR | LinJ.36.6110  (*KH1*) | 13 | rt_*KH1*_36.6110_Fw | CCTCCTCGCAACAACTTCA | 58,4 | 105 |
|  |  | 14 | rt_*KH1*_36.6110_Rev | GTCACGGTTACAGTTGGGATAG | 57,1 |  |
|  | LinJ.36.2480 (GAPDH) | 15 | rt_GAPDH_Fw | TGTGGAGAAGCGAGCAACGTACAA | 67 | 74 |
|  |  | 16 | rt_GAPDH_Rev | ATGAGACGAGCTTGACGAAGTGCT | 65 |  |
|  | LinJ.16.1640  (DNA polimerase) | 17 | rt_DNApol_Fw | CGAGGGCAAGACATAC | 48 | 69 |
|  |  | 18 | rt_DNApol_Rev | GAGAGCGGGCACCAATCAC | 62 |  |
| *Southern blot* | 5’UTR-*KH1* | 19 | 5’UTR-*KH1*-Fw | CCGTCTATCGCTTTTACTTCG | 58 | 364 |
|  |  | 20 | 5’UTR-*KH1*-Rev | CGTGGAAGTGCTCGAGACGGC | 65.7 |  |
|  | *KH1* | 21 | KH1-Fw | ATGACGCAGGAAACCTCCCCT | 61.8 | 859 |
|  |  | 21 | *KH1*-Rev | GCTTGAACGGCTGTGGCTTCT | 61.8 |  |

**Supplementary Table S1****. Primers and Probes –** The underlined sequences correspond to the restriction sites inserted in the amplification products. The gray sequences correspond to the homology sequences required for carrying out the fusion PCR. Primers were designed using the Primer Quest® tool (www.idtdna.com/Primerquest/Home/Index).

NC

CL1

CL3

CL2

CL5

CL4

CL6

WT

1.65 -

**NEO**

**3’ *UTR***

**OUT**

1600 bp

**5’ *UTR***

**OUT**

MW

**a**


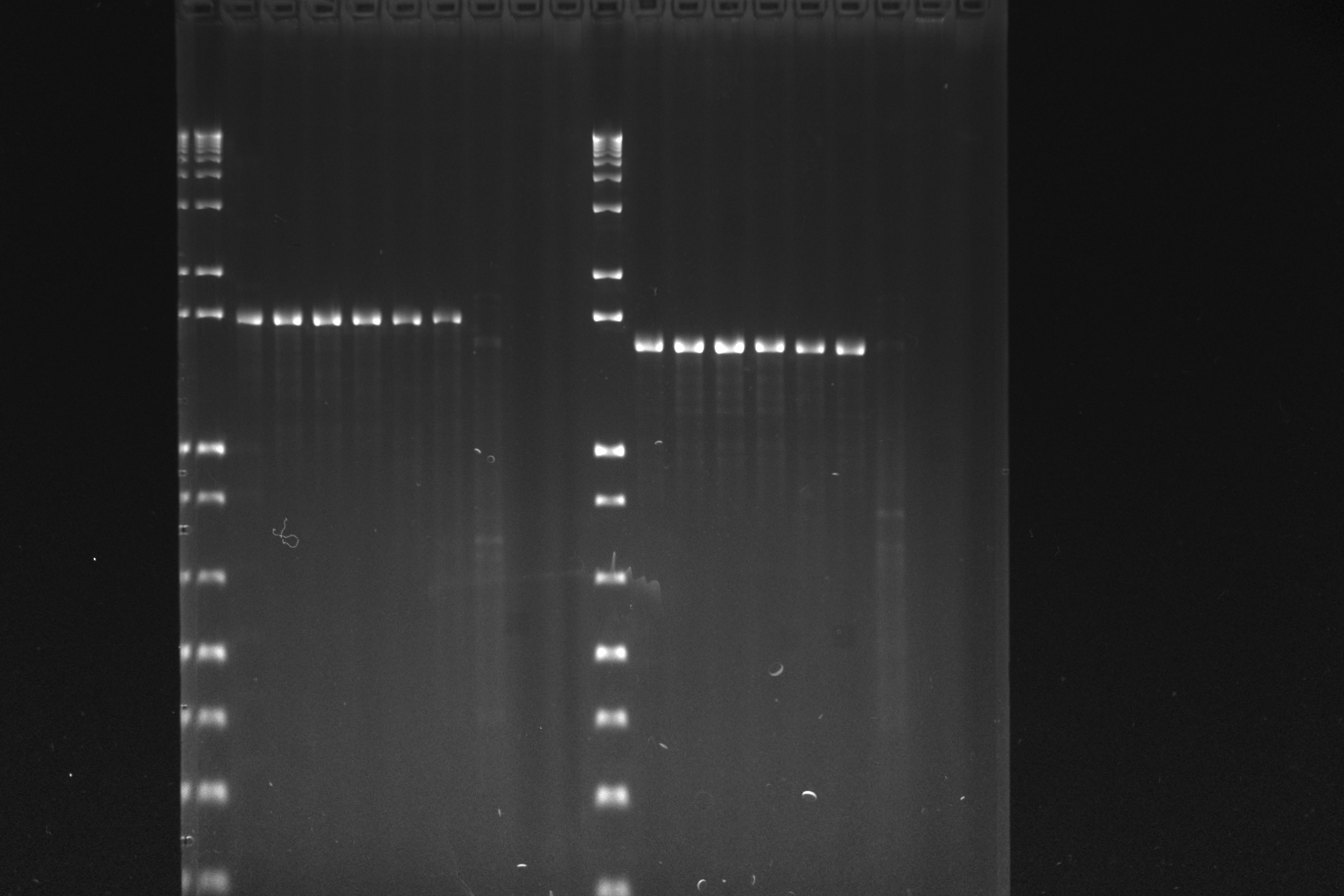


kb


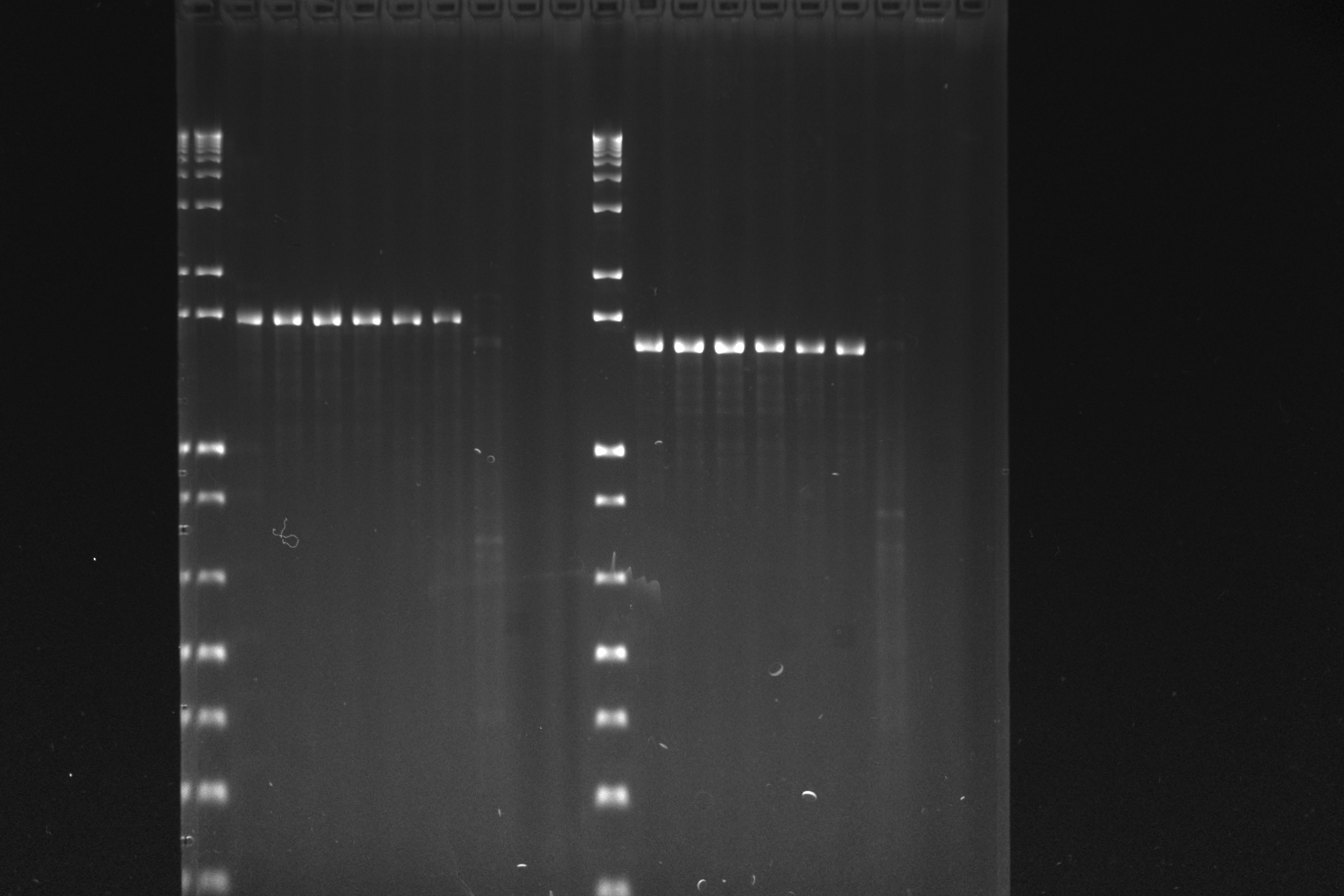


CL1

CL3

CL2

CL5

CL4

CL6

WT

NC

**HYG**

**3’ *UTR***

**OUT**

1476 bp

**5’ *UTR***

**OUT**

MW

**b**

1.65 -

kb

**Supplementary Figure S1. The deletion of *KH1* gene in *L. infantum* was accessed by two successive rounds of homologous gene replacement –** A) In the first round, one copy of *KH1* gene was replaced by *NEO* cassette and the replacement was visualized by PCR amplification using a primer that anneals within the *NEO* cassette sequence, and a primer located on a *KH1* chromosomal flanking sequence, outside the targeting fragment on the 5′. The scheme below the gel shows the expected size of the PCR products. B) In the second round, the Δ*Likh1^+^* heterozygote was transfected with the *HYG* cassette for replacement at the second copy of *KH1*. Homologous replacement was visualized by PCR amplification using a primer that anneals within the *HYG* cassette sequence, and a primer located on a KH1 chromosomal flanking sequence, outside the targeting fragment on the 5′. After the second round, the transfectants presented both *NEO* and *HYG* fragments with expected length, suggesting that both cassettes were correctly integrated. MW, molecular weight; WT, wild-type; NC, negative control with no DNA template; CL1-Cl6, independent clones of *L. infantum* Δ*Likh1.* Here we show region of interest cropped from original images available at the end of supplementary material.


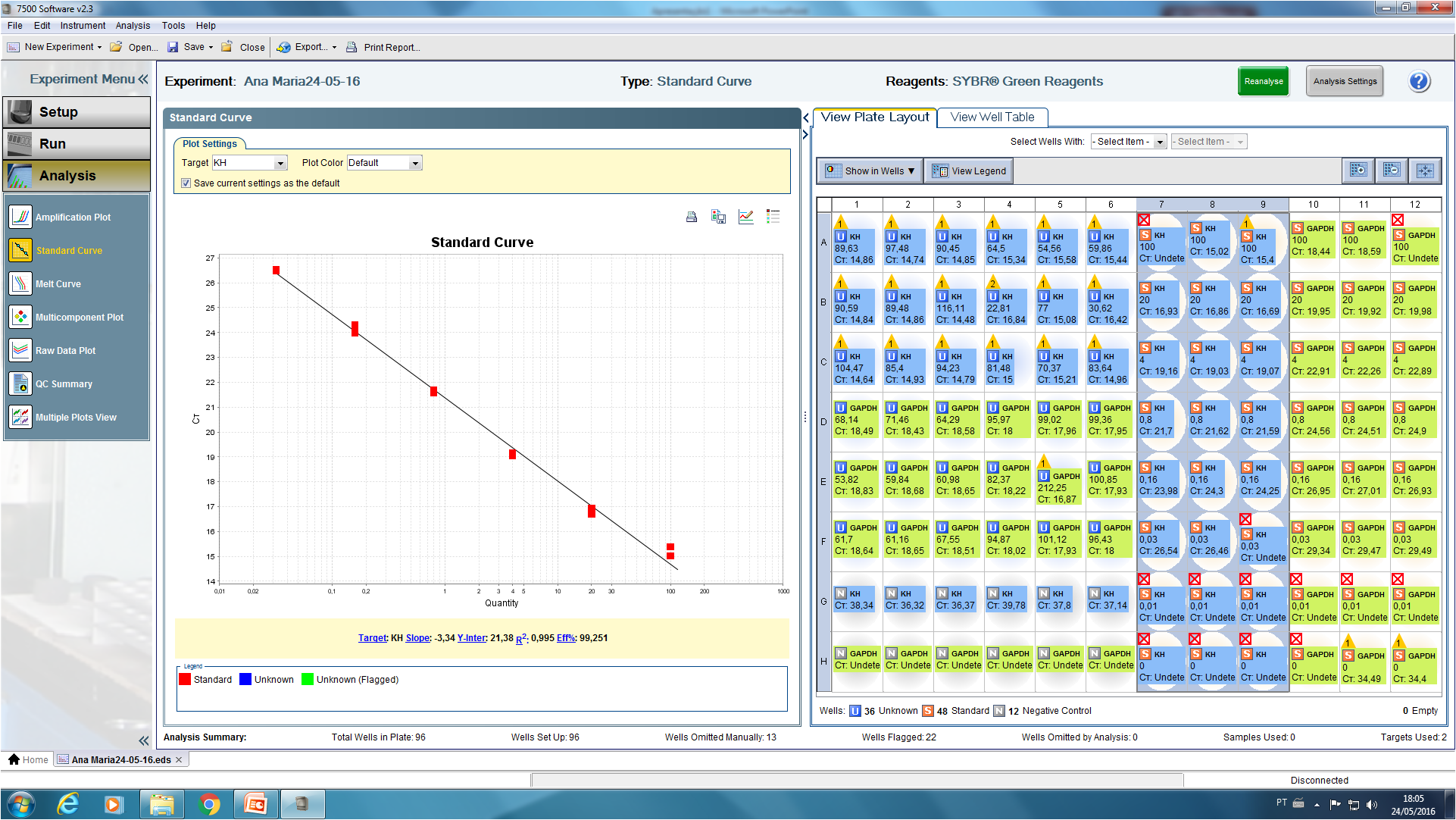

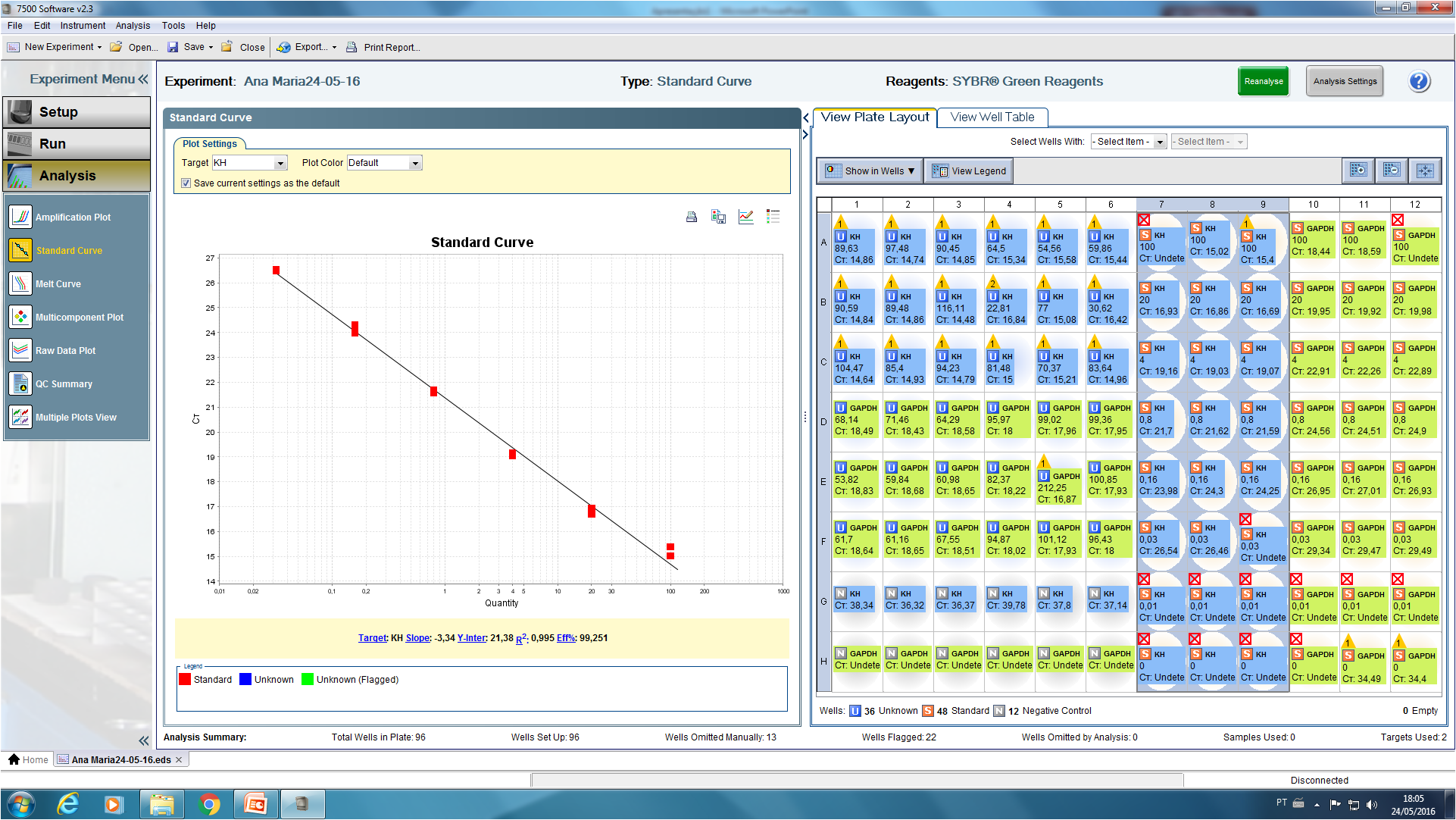


**a**

**b**


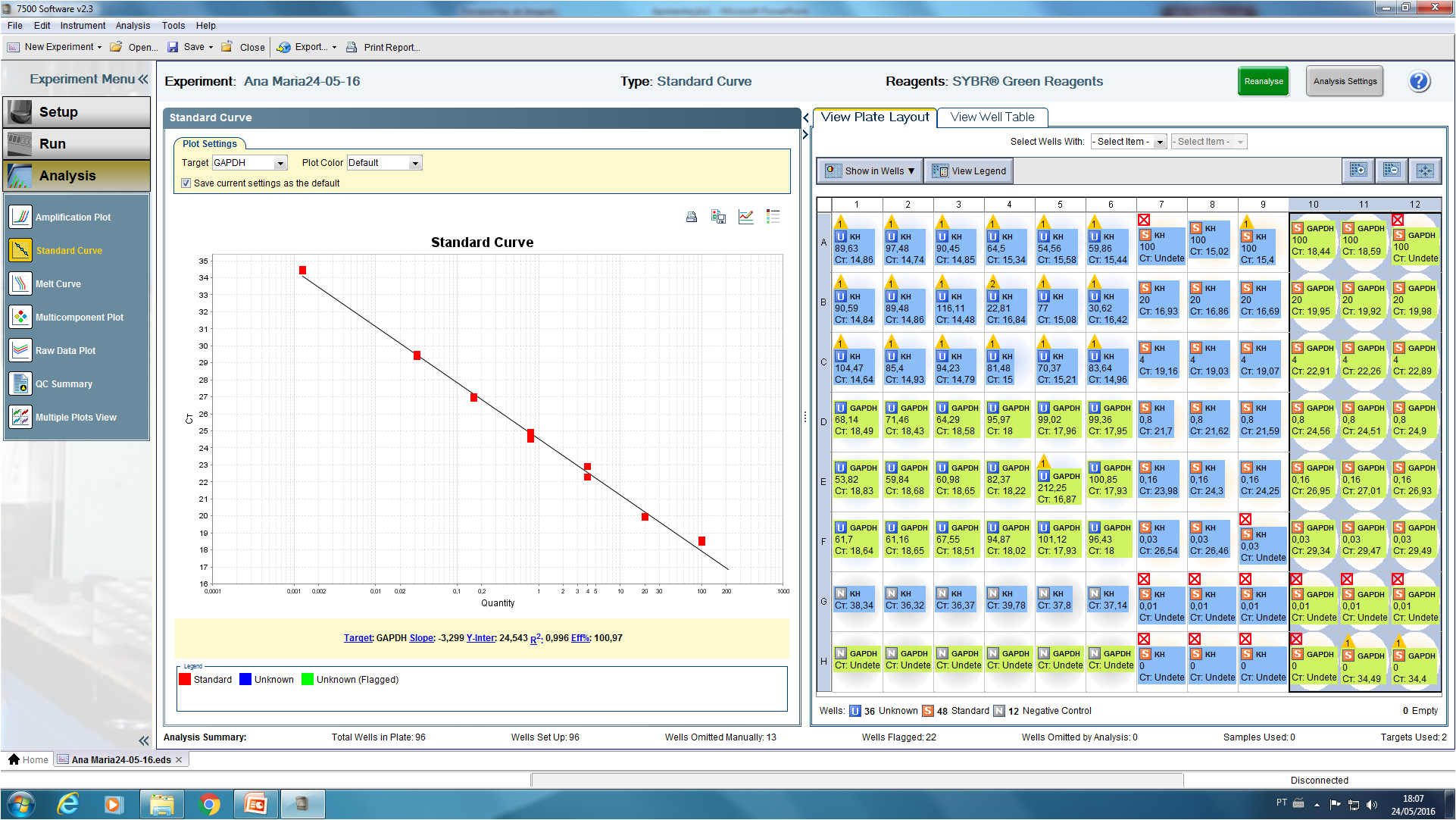

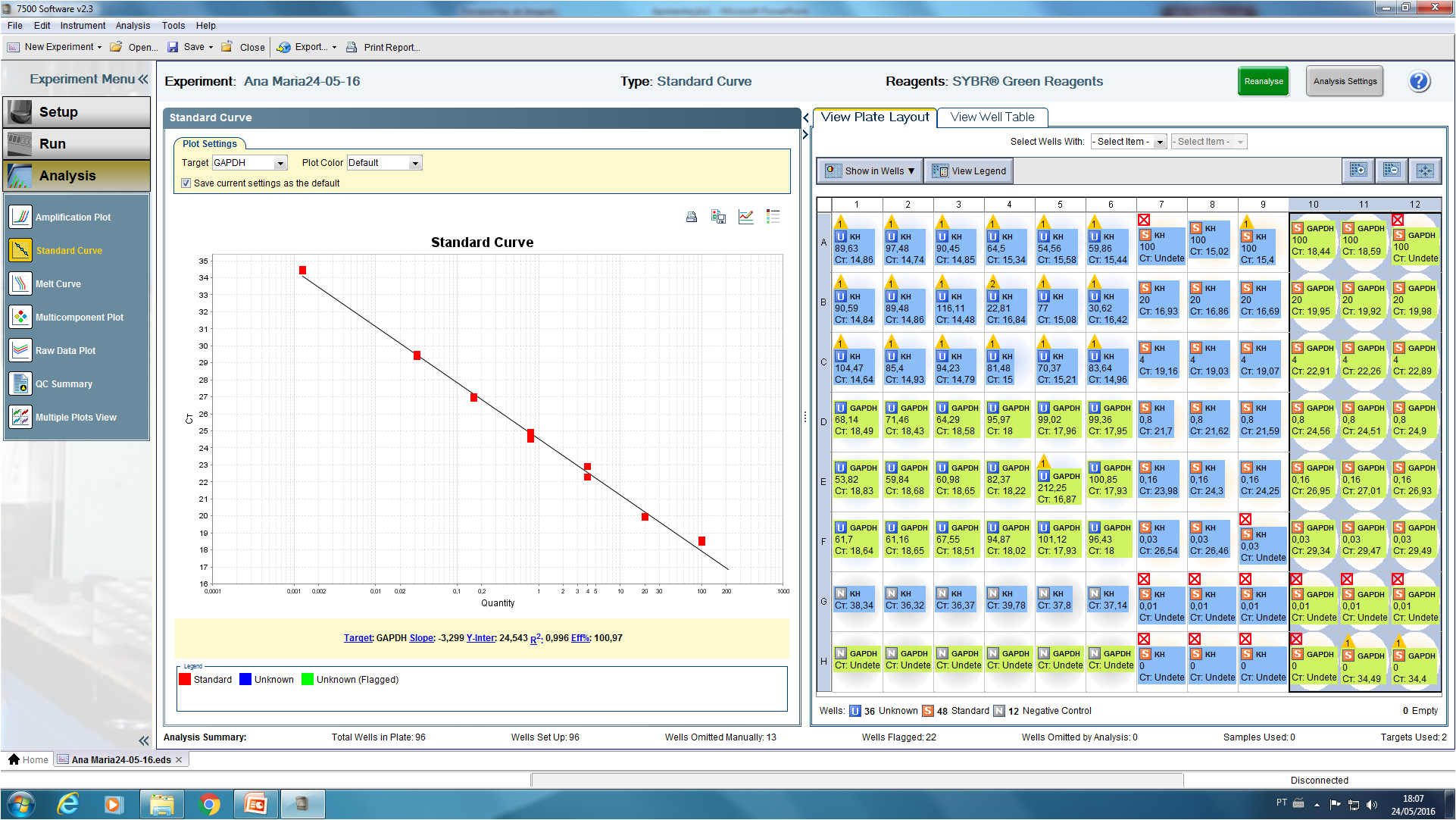


**c**


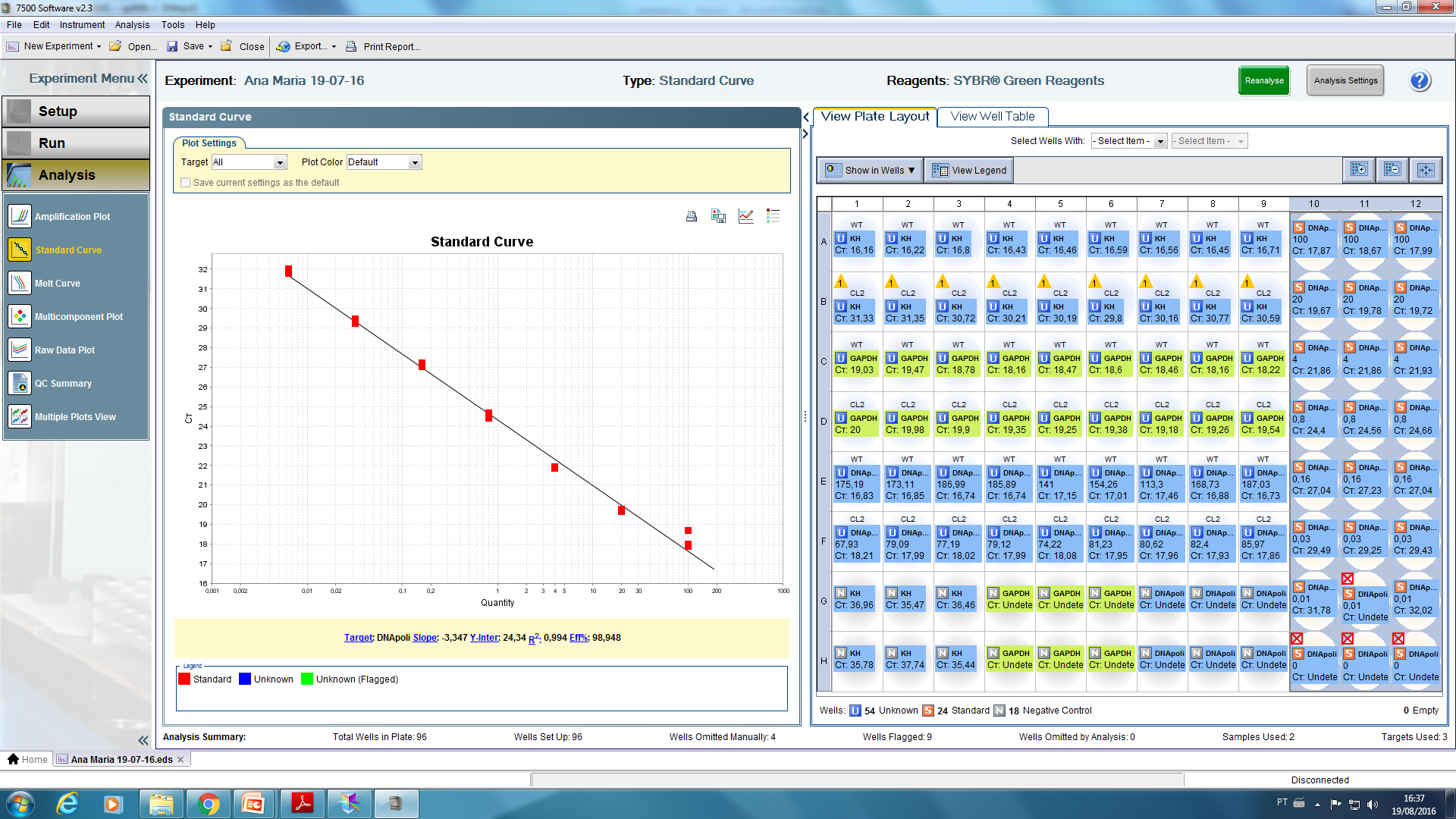

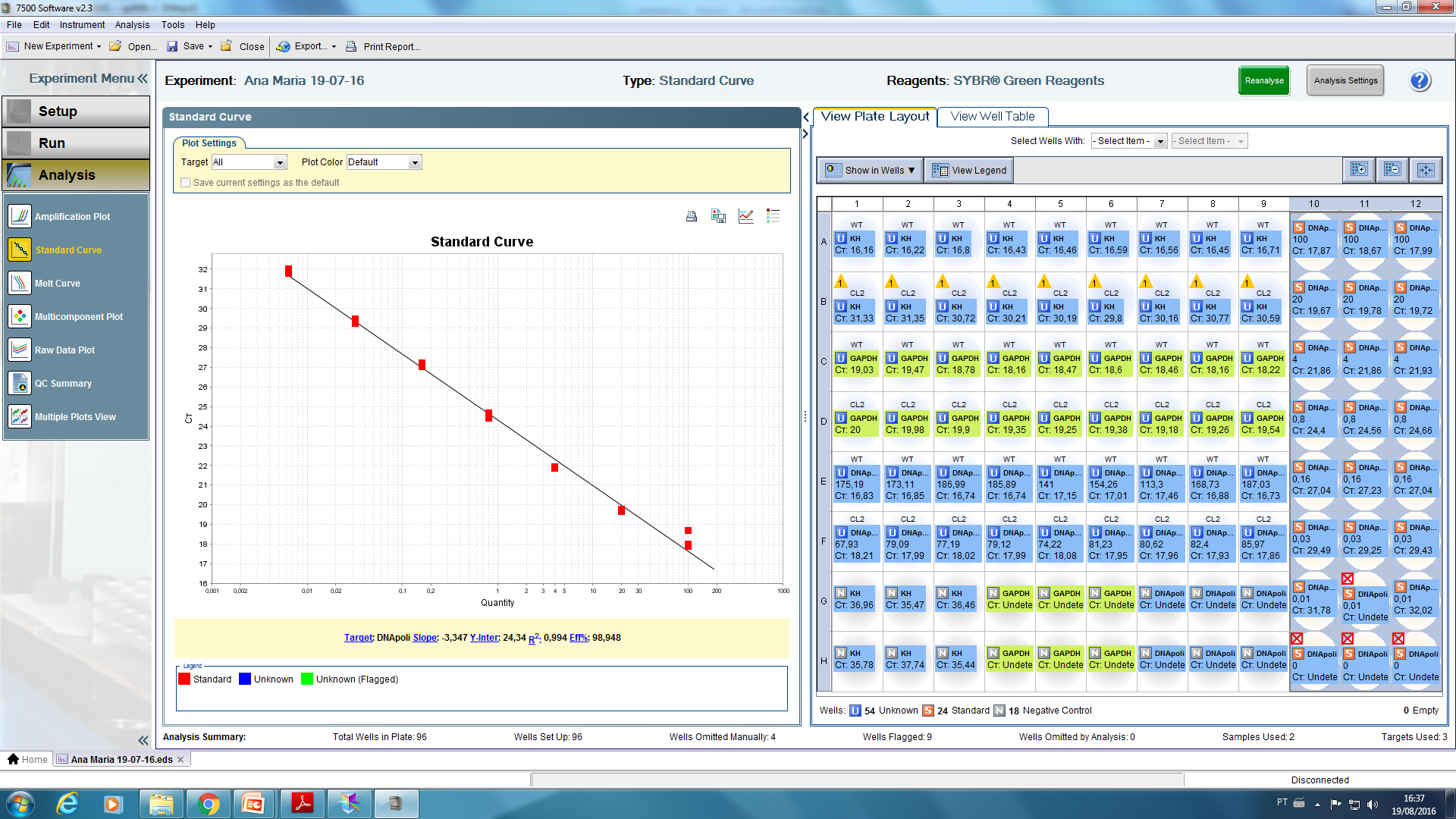


**Supplementary Figure S2. Standardization of qPCR –** Standard curves of *KH1* (A), *GAPDH* (B) and *DNA polymerase* (C) genes, showing the amplification efficiencies for each pair of primers.

**Supplementary Figure S3. Glucose uptake by *L. infantum* WT and Δ*Likh1* null mutant promastigotes forms –** Wild-type and Δ*Likh1* parasites (1x10^8^ parasites/mL) were washed, resuspended in PBS (140 mM NaCl, 2.7 mM KCl, 8.1 mM Na2 HPO4 ) and incubated at 37°C with 90 μM of the fluorescent analog of glucose, 2-NBDG (*2-(N-(7-Nitrobenz-2-oxa-1,3-diazol-4-yl)Amino)-2-Deoxyglucose*) (Invitrogen™). As a control, the parasites were also incubated with 2 mM glucose prior to the incubation with 2-NBDG. After the incubations (1 h or 3 h), the parasites were washed to remove any 2-NBDG that was not incorporated. The samples were lized with Tween-20 and then evaluated on SpectraMax M5 spectrofluorimeter (Molecular Devices) for fluorescence intensity at wavelengths of 488/560 nm of excitation/emission, respectively. Control (background): promastigote fluorescence background without 2-NBDG. Experiments were performed twice in triplicate. Statistical analysis was performed using the 1way ANOVA test with Bonferroni post-test (*P ≤ 0.05; **P ≤ 0.01; ***P ≤ 0.001).

**Supplementary Figure S4. Evaluation of susceptibility of Δ*Likh1* mutants to antimony –** Promastigotes of *L. infantum* lines: wild-type (WT), heterozygous knockout (Δ*Likh1^+^*), and null mutant (Δ*Likh1*) were washed, counted and 2x10^6^ parasites/mL were incubated in the presence of different concentrations of antimony potassium tartrate (Sigma), at 26 ° C for 48 h. The number of parasites that grown in the presence and absence of the drug was determined using the *Z1 Coulter^®^ Particle Counter* (Beckman Coulter™). The concentration that inhibits 50% growth (IC_50_) was determined by non-linear regression using the program GraphPad Prism, Inc.

**Supplementary Figure (S5). – Humoral response induced by immunization with Δ*Likh1* by different administration routes.** Δ*Likh1* SLA-specific IgG_total_ and IgG_1_ and IgG_2a_ subclasses were measured by conventional ELISA on Balb/c mice serum, 20 days after the last immunization. Data are expressed as the mean and standard deviation of the Absorbance at 492 nm obtained with sera, at a dilution of 1:150, of at least 6 animals per group. ***p <0.0001 and *p <0.01, One-Way-ANOVA followed by Bonferroni’s multiple comparisons test.

**Gels and blots (original images)**

Supplementary Figures S1a and S1b





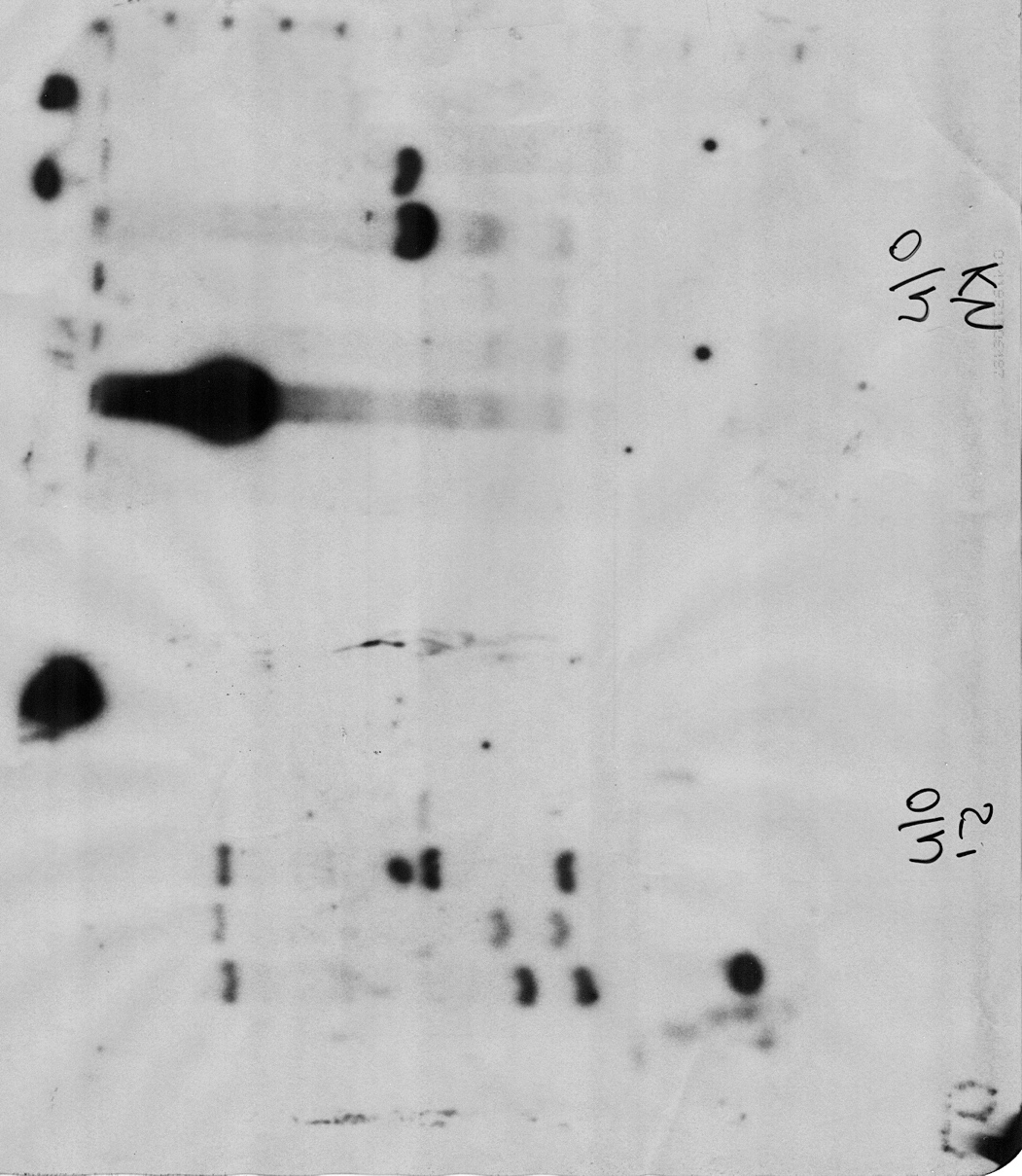


**Figures 1c and 1d**


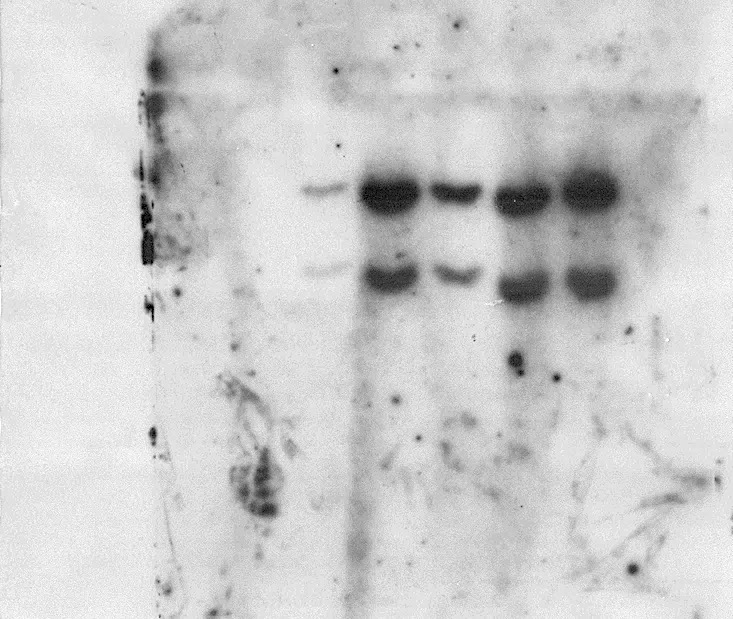


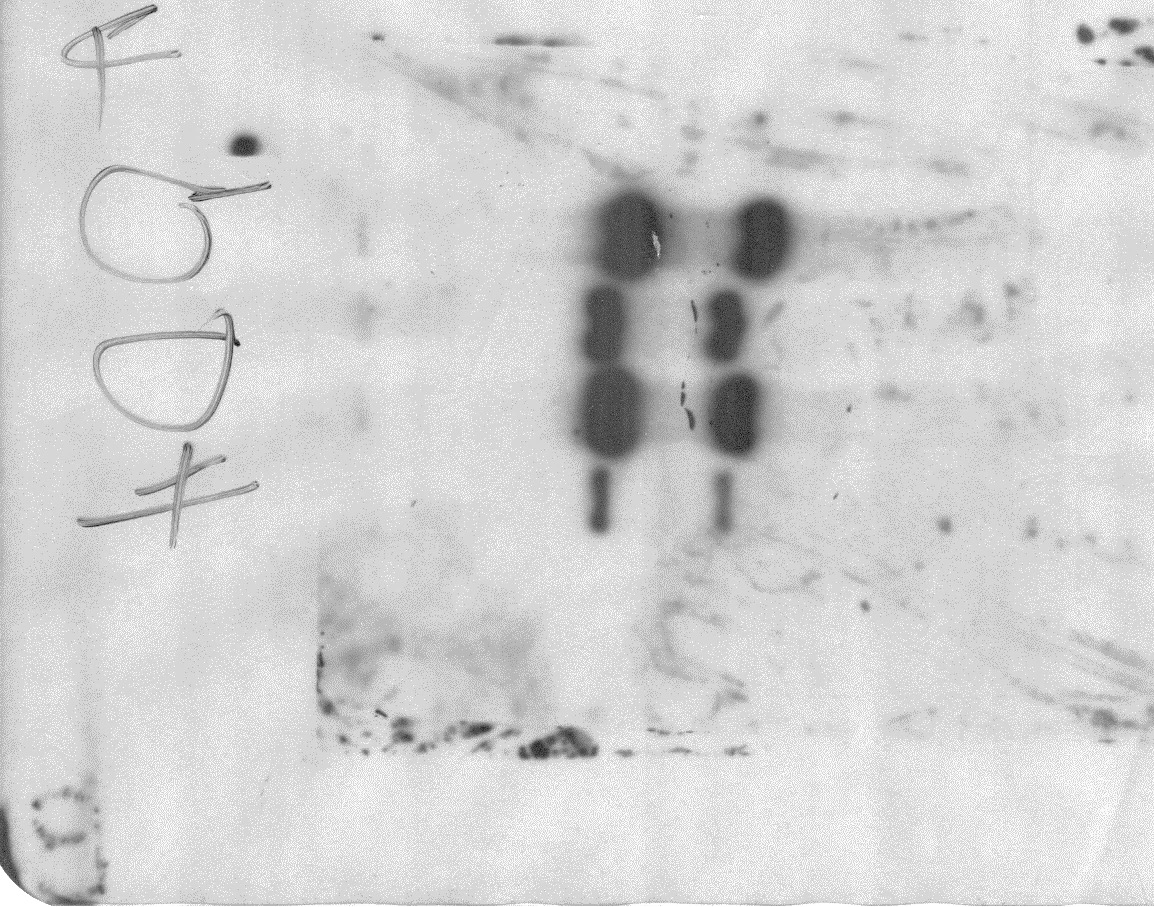

Supplement: Supplementary file 1 — Supplementary Information [file 41598_2018_30076_MOESM1_ESM.docx]
